# Intersecting impact of CAG repeat and huntingtin knockout in stem cell-derived cortical neurons

**DOI:** 10.1016/j.nbd.2025.106914

**Published:** 2025-04-19

**Authors:** Jennifer T. Stocksdale, Matthew J. Leventhal, Stephanie Lam, Yu-Xin Xu, Yang Oliver Wang, Keona Q. Wang, Reuben Thomas, Zohreh Faghihmonzavi, Yogindra Raghav, Charlene Smith, Jie Wu, Ricardo Miramontes, Kanchan Sarda, Heather Johnston, Min-Gyoung Shin, Terry Huang, Mikelle Foster, Mariya Barch, Naufa Amirani, Chris Paiz, Lindsay Easter, Erse Duderstadt, Vineet Vaibhav, Niveda Sundararaman, Dan P. Felsenfeld, Thomas F. Vogt, Jennifer Van Eyk, Steve Finkbeiner, Julia A. Kaye, Ernest Fraenkel, Leslie M. Thompson

**Affiliations:** a Department of Neurobiology and Behavior, UC Irvine, Irvine, CA 92677, USA; b MIT PhD Program in Computational and Systems Biology, Cambridge, MA 02139, USA; c MIT Department of Biological Engineering, Cambridge, MA 02139, USA; d Department of Biological Engineering, Massachusetts Institute of Technology, Cambridge, MA 02139, USA; e Center for Systems and Therapeutics, Gladstone Institutes, San Francisco, CA 94158, USA; f Advanced Clinical Biosystems Research Institute, Smidt Heart Institute, Cedars-Sinai Medical Center, Los Angeles, CA 90048, USA; g Institute of Data Science and Biotechnology, Gladstone Institutes, San Francisco, CA 94158, USA; h Department of Psychiatry and Human Behavior, UC Irvine, Irvine, CA 92697, USA; i Department of Biological Chemistry, UC Irvine, Irvine, CA 92697, USA; j Institute for Memory Impairments and Neurological Disorders, UC Irvine, Irvine, CA 92697, USA; k CHDI Management, Inc, New York, NY 10001, USA; l Department of Physiology, University of California, San Francisco, San Francisco, CA 94158, USA; m Department of Neurology, University of California San Francisco, San Francisco, CA 94158, USA; n Taube/Koret Center for Neurodegenerative Disease Research, Gladstone Institutes, San Francisco, CA 94158, USA

**Keywords:** Huntington’s disease, Embryonic stem cells, RUES2, Multi-omics, Robotic microscopy, Machine learning, Features, Network analysis

## Abstract

Huntington’s Disease (HD) is caused by a CAG repeat expansion in the gene encoding huntingtin (HTT). While normal HTT function appears impacted by the mutation, the specific pathways unique to CAG repeat expansion versus loss of normal function are unclear. To understand the impact of the CAG repeat expansion, we evaluated biological signatures of HTT knockout (*HTT* KO) versus those that occur from the CAG repeat expansion by applying multi-omics, live cell imaging, survival analysis and a novel feature-based pipeline to study cortical neurons (eCNs) derived from an isogenic human embryonic stem cell series (RUES2). *HTT* KO and the CAG repeat expansion influence developmental trajectories of eCNs, with opposing effects on growth. Network analyses of differentially expressed genes and proteins associated with enriched epigenetic motifs identified subnetworks common to CAG repeat expansion and *HTT* KO that include neuronal differentiation, cell cycle regulation, and mechanisms related to transcriptional repression, and may represent gain-of-function mechanisms that cannot be explained by *HTT* loss of function alone. A combination of dominant and loss-of-function mechanisms are likely involved in the aberrant neurodevelopmental and neurodegenerative features of HD that can help inform therapeutic strategies.

## 1. Introduction

Huntington’s disease (HD) is an autosomal dominant brain disorder that affects approximately 10 in every 100,000 individuals (McColgan and Tabrizi, 2018). HD typically manifests clinically at midlife; however, juvenile HD cases also occur and display more severe clinical manifestation and faster disease progression (Bakels et al., 2022). HD is caused by an unstable CAG repeat expansion in exon 1 of the *Huntingtin* (*HTT*) gene (The Huntington’s Disease Collaborative Research Group, 1993) resulting in an expanded polyglutamine repeat (polyQ)-containing HTT protein. The length of the repeat expansion is highly polymorphic and in general correlates with disease severity: individuals with fewer than 36 repeats are unaffected, those with 36–39 repeats have variable penetrance (Langbehn et al., 2004), adult-onset HD occurs in individuals with 40–60 repeats, and juvenile-onset HD typically occurs in individuals with more than 60 repeats (Fusilli et al., 2018). The hallmark clinical features of HD are the progressive loss of physical and mental functions characterized by uncontrolled movements, and psychiatric and cognitive impairment (McColgan and Tabrizi, 2018). The mutation causes dysfunction and death of medium spiny neurons in the striatum and significant dysfunction and atrophy of the cortex. During the past three decades, great progress has been made in understanding mechanisms involved in HD pathogenesis (Jimenez-Sanchez et al., 2017; Tabrizi et al., 2020), which have primarily centered around a dominant gain of aberrant function, and in identifying genetic modifiers of disease onset, which to date are largely functionally related to DNA mismatch repair (Goold et al., 2019; Genetic Modifiers of Huntington’s Disease, C, 2015; Ferguson and Tabrizi, 2023; Pinto et al., 2013).

There has also been significant progress in understanding normal HTT function, potentially as a protein scaffold for processes such as autophagy (Ochaba et al., 2014; Rui et al., 2015), vesicular transport (Vitet et al., 2020), neurodevelopment (van der Plas et al., 2020; Ratie and Humbert, 2024), and a broad range of other cellular processes (reviews (Liu and Zeitlin, 2017; Saudou and Humbert, 2016; Wright et al., 2017)). While loss of HTT function is not sufficient to cause HD, given that genetic mutation or deletion of *HTT* alleles in human disorders including Wolf-Hirschhorn Syndrome (Battaglia et al., 2015) do not cause a neurodegenerative disease, the CAG repeat expansion does cause an apparent disruption of normal HTT functions, including synapse formation and altered neurodevelopment among other disrupted processes (Gauthier et al., 2004; McKinstry et al., 2014; Ruzo et al., 2018; Martinez-Vicente et al., 2010; Braz et al., 2022). Further, homozygous CAG repeat expansion does not result in consistently worse disease than a heterozygous mutation in human HD (Cubo et al., 2019; Wexler et al., 1987), reflecting the dominant nature of the mutation, whereas total loss of HTT is embryonic lethal in mice (Nasir et al., 1995). Several phenotypes impacted by expanded repeat HTT and HTT knockout (KO) are overlapping, including those impacting neurodevelopment and chromosomal stability (McKinstry et al., 2014; Ruzo et al., 2018; Nasir et al., 1995; Auerbach et al., 2001; O’Kusky et al., 1999). However, to date, there has not been a systematic investigation of the degree to which HD-associated neuronal molecular and morphological changes are due to CAG repeat expansion versus loss of HTT function.

To address this, isogenic embryonic stem cell (ESC) lines with normal, CAG-expanded and a homozygous deletion of *HTT* exon 1, resulting in loss of the HTT protein expression (*HTT* KO), were differentiated to generate cortical-like neurons (eCNs) and a comprehensive analysis using live cell imaging and multi-faceted OMICs was conducted. We chose to investigate a homozygous deletion versus a heterozygous deletion to more fully understand the function of HTT. The premise for using total KO is because the mutant protein can still confer some of the normal functions of HTT (Duyao et al., 1995; Zeitlin et al., 1995; White et al., 1997). Total HTT KO also enables a clear assessment of loss-of-function (LOF) in the absence of any normal or mutant HTT, as a full KO will avoid any complicating factors when evaluating the overlap between aberrant, gain-of-function changes in the presence of the mutation and LOF in the absence of any HTT protein.

Previous time-lapse imaging using robotic microscopy (RM) of live neurons patterned from induced pluripotent stem cells (i-neurons) from HD patients and controls demonstrated neurodegenerative phenotypes, including increased cell death, greater susceptibility to trophic factor withdrawal, and differences in neurite length (HD iPSC Consortium, 2012; HD iPSC Consortium, 2017). To ascertain cellular changes directly attributable to the CAG repeat expansion, we utilized isogenic expanded CAG repeat lines and report a subset of similar findings as previous studies in HD i-neurons (HD iPSC Consortium, 2012; HD iPSC Consortium, 2017). Interestingly, we do not observe similar changes in *HTT* KO lines, suggesting that these phenotypes are specifically due to a gain-of-function mechanism driven by the polyglutamine expansion.

We also developed a novel platform for image-based feature classification, commonly used in machine learning (ML) applications (Tang et al., 2014), as a way to assess dynamic morphologic changes over time. Previously, ML called a deep neural network (DNN) was used to build a classifier to discriminate CAG-expanded and *HTT* KO from controls on images from fixed and stained cortical organoids containing clusters of cells (Metzger et al., 2022). We wanted to harness the power of ML, but at a single cell resolution and over time. To do this, we subjected images of live eCNs to feature extraction and object recognition algorithms (Canny, 1986; GitHub, 2023; Harris et al., 2020; Kaso, 2018; Li et al., 2016; Uppala and Sahr, 1997; van der Walt et al., 2014; Virtanen et al., 2020) that capture a range of engineered features commonly used in shallow ML approaches (McQuin et al., 2018; Trier et al., 1996). We analyzed how these features evolved over time as a proxy for monitoring dynamic cellular changes during development. Our results highlight those that are distinct versus overlapping morphologic alterations in response to the CAG expansion compared to the *HTT* KO.

Further, we integrated this multi-omics data with our morphological analysis and discovered specific phenotypes contributed by *HTT* KO and CAG repeat expansion. Both *HTT* KO and expansion caused neurodevelopmental changes, however these comprise features and pathways that are both overlapping and distinct. By identifying key molecular drivers of these phenotypes and separating out pathways that are unique to KO versus CAG expansion, our data have important implications for future therapeutic development for HD.

## 2. Results

### 2.1. Cortical neuron differentiation and cell-type characterization across HTT CAG repeat and KO RUES2 lines

**Fig. 1.**
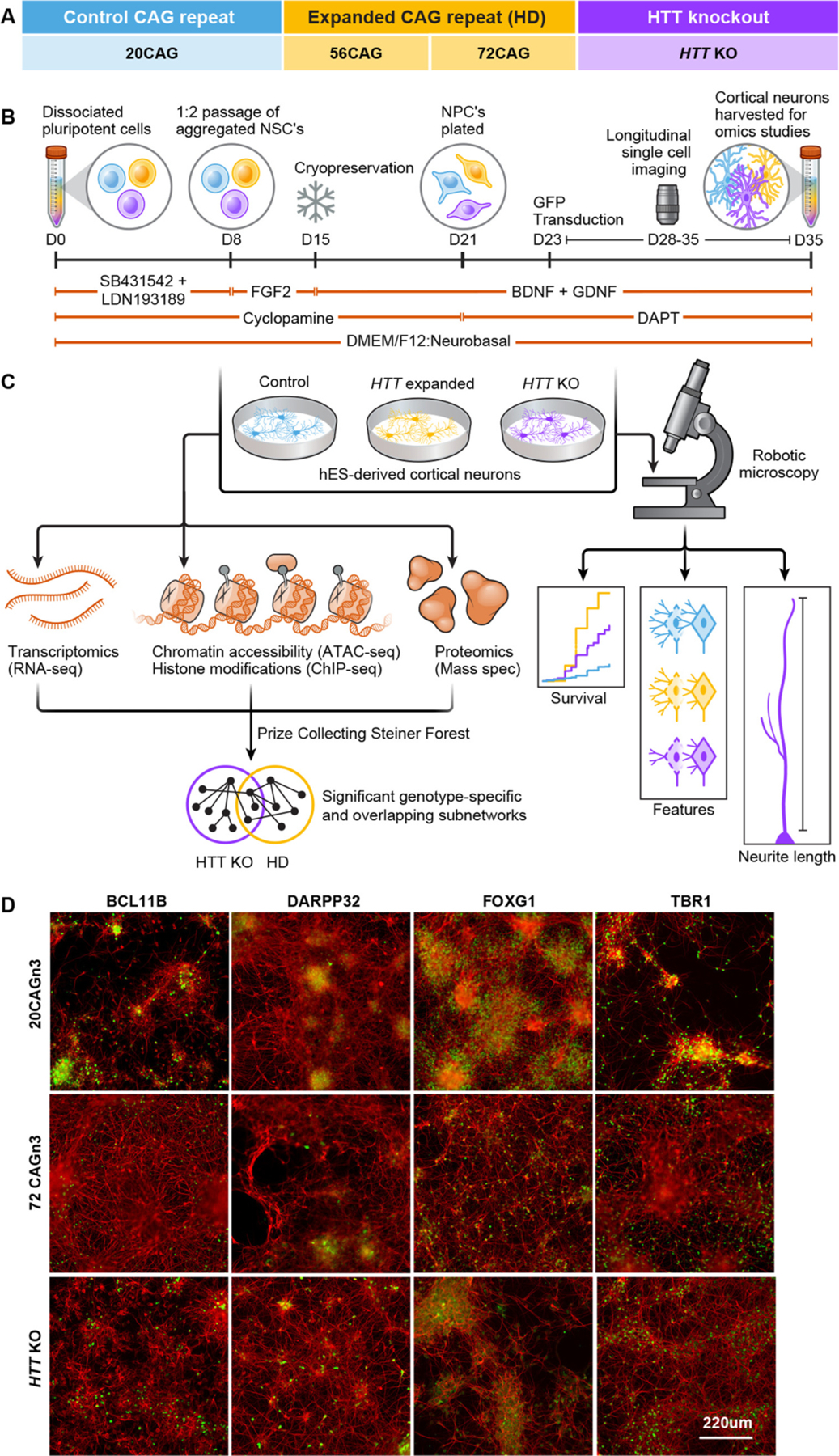
Methodological overview and study design. (A) Table of isogenic cell lines used in this study. A complete list of cell lines can be found in Supplemental Table 1 (B) Schematic overview of the cortical neuron differentiation depicting the timeline of study measurements. The top bar denotes the differentiation timeline, bottom bars delineate the small patterning molecules used during each period of the protocol. (C) Schematic overview showing differentiation into cortical neurons (eCNs) used for robotic imaging (RM) analysis as well as generation of the 4 different omics assays (RNAseq, ATACseq, ChIPseq, and mass spectrometry), and integration of all data sets to reveal dysregulated pathways using Omics Integrator. D) Representative immunofluorescence images showing expression of neuronal markers in the eCNs that were subjected to RM and fixed at differentiation day ~35. Expression of BCL11B, DARPP32, FOXG1, Ki67 and TBR1 in HTT KO, 20CAG and 72CAG eCNs. CTIP2, DARPP32, FOXG1 and TBR1 (green) were co-stained with MAP2 (red) colocalized with nuclei (blue). scale bar = 220 μm.

To better understand the cellular consequences of the *HTT* CAG repeat expansion versus loss of HTT function, we employed a systems approach using multi-acquisition omics analysis including ATAC-seq, ChIP-seq, RNA-seq and proteomics in conjunction with cellular phenotyping analyses (Fig. 1A–C). To eliminate the patient-to-patient and cell line-to-cell line variability which complicates differential analysis, we applied these analyses to a previously reported isogenic series developed in the RUES2 ESC line (Ruzo et al., 2018). The series consists of control clonal lines with normal CAG repeat lengths (20CAGn1, 20CAGn2, 20CAGn3, and 20CAGn4), an expanded line harboring 56 CAG repeats (56CAG), three clones of a juvenile-onset CAG expansion containing 72 CAG repeats (72CAGn1, 72CAGn2, 72CAGn3), and one line with homozygous deletion of *HTT* exon 1 resulting in loss of the HTT protein expression (*HTT* KO) (Ruzo et al., 2018) confirmed by western blot (Fig. 1A for repeats, Supplemental Fig. 1 for western, and Supplemental Table 1 for clone designations). These lines were differentiated down a cortical neuron lineage to generate eCNs using a recently developed protocol based in part on Shi et al. 2012 (Shi et al., 2012) with the addition of a Sonic hedgehog (SHH) inhibitor, cyclopamine (Cao et al., 2017), to direct away from a medial ganglionic patterning (Conforti et al., 2020), and extended FGF2 signaling to enhance the derivation of glutamatergic cortical neurons. Maturation was accelerated by using the gamma secretase inhibitor DAPT, which forces cells to exit the cell cycle (Telezhkin et al., 2016) (Fig. 1B). After ~35 days of differentiation, these eCN populations consist of a large portion of MAP-2 positive signaling area as well as cells expressing the forebrain marker FOXG1, early cortical lineage marker (layer VI) TBR1, and a small number of cells positive for cortical layer V marker BCL11B (Fig. 1D and Supplemental Fig. 2).

We examined the differentiation efficiency of each line by staining for cell-specific markers at differentiation day ~35. eCNs were fixed, stained and quantified to ascertain the percentage of positively labeled cells for each antibody using a modified Cell Profiler pipeline as previously described (Wang et al., 2024) (Supplemental Table 2, 3). All clones (20CAGn3/n4, 72CAGn1/n2/n3 and *HTT* KO) of eCNs patterned to contain ~60 % FOXG1 and ~30 % TBR1 positively expressing cells (Supplemental Fig. 3 A–D). We did not detect a significant difference in the numbers of these cell types across all clones. These cultures developed a small proportion (~10 %) of inhibitory GABAergic neurons as identified by neurons expressing DARPP-32. There was a subtle, but significant reduction of the number of DARPP-32 positive cells in the *HTT* KO compared to the 20CAG eCNs (Supplemental Fig. 3E, F). We also observed a small proportion (~10 %) of BCL11B positive cells, with the *HTT* KO displaying slightly lower number of positively labeled cells compared to control eCNs (Supplemental Fig. 3G, H) and a small portion of persistent proliferating cells, as identified by the Ki-67 marker (Supplemental Fig. 3I, J).

### 2.2. Expanded repeat and KO eCNs display differences in survival, neurite length and morphological differences over time compared to controls

**Fig. 2.**
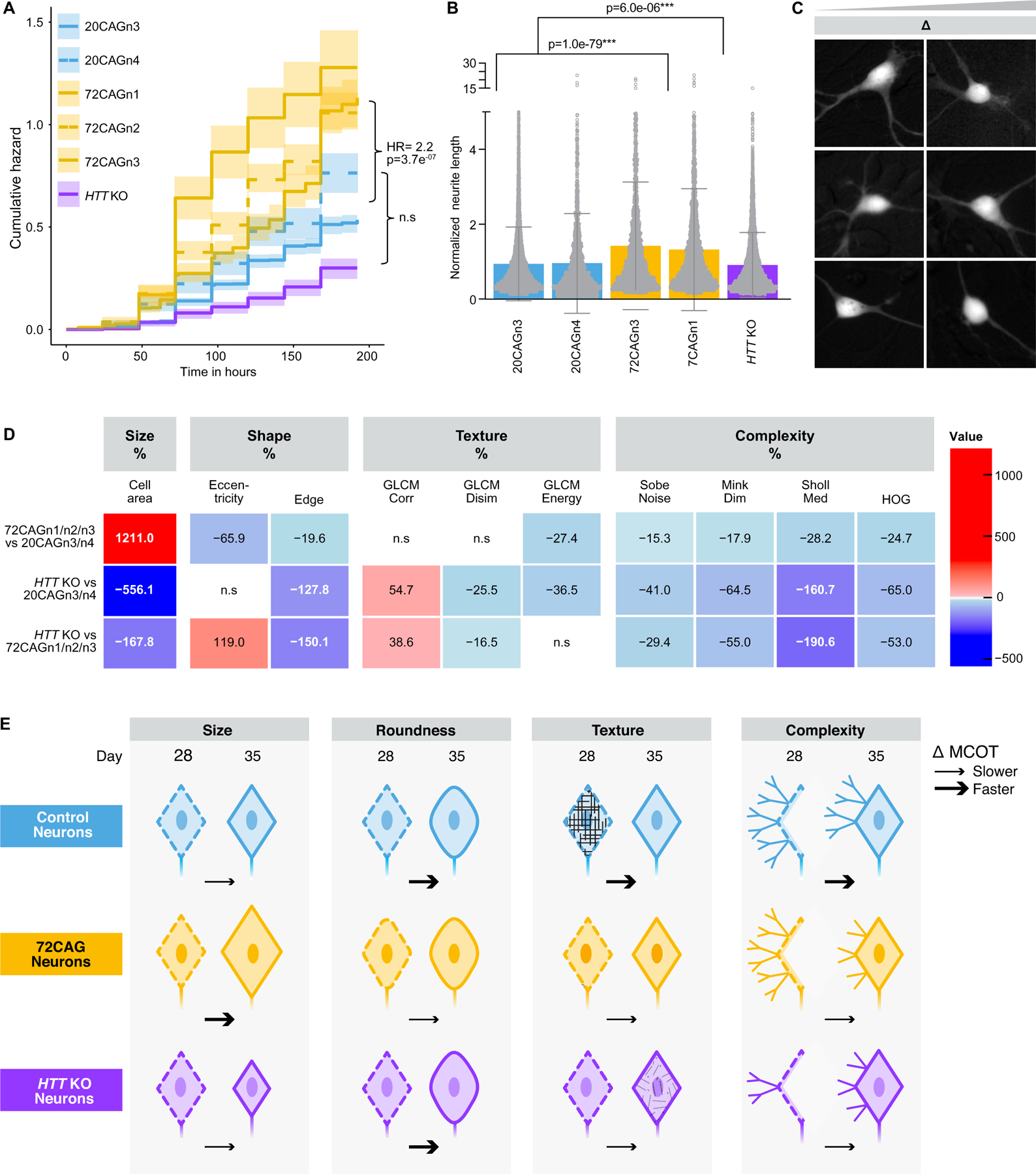
Live cell imaging of eCNs shows spontaneous degeneration and morphological changes between CAG-expanded HTT knockout and control eCNs. differences in neurite length across *HTT* genotypes. (A) eCNs were transduced with LV-Synapsin-EGFP and tracked for 7 days as shown in example images in supplemental Fig. 4. Using manual curation, cells were determined live or dead, and the cumulative death rate (live/dead cells) was plotted over time as previously described. A Cox mixed effects model (Therneau, 2020) was used to calculate hazard ratio of 72CAG eCNs cells to controls, HR = 2.12 with p-value of 3.7e-07 ***. There was no difference between control eCNs and KO eCNs, HR = 1.2, p=0.43. 20CAGn3 = 1432 neurons; 20CAGn2 = 515 neurons; 72CAGn1 = 575 neurons, 72CAGn2 = 972 neurons; 72CAGn3 = 340 neurons, *HTT* KO = 555 neurons, 9 experiments. (B) Neurites were measured using CellProfiler to capture neurite length on images acquired by RM differentiation day ~32. Neurite values were normalized as previously described (HD iPSC Consortium, 2017). We used a LMM (Shin et al., 2022) to compare groups. Neurites from 72CAG eCNs are longer than control and KO eCNs, $p=1.0{\text{e}}^{-79}$ whereas neurites from the HTT KO are shorter than control eCNs $p=6.0{\text{e}}^{-06}$ 20CAGn3 = 10,849 neurons, 20CAGn4 = 2725 neurons, 72CAGn3 = 2320 neurons; 72CAGn1 = 4092 neurons, *HTT* KO = 9691 neurons, from 11 experiments. (C) Representative images show changes in cortical neuronal morphology over time for the 20CAG, 72CAG and *HTT* KO genotypes. (D) Time interaction feature analysis reveals dynamic morphological changes in CAG expanded and *HTT* knockout eCNs. Heatmap showing changes across the different groups over time. A time interaction model was used to determine the change in feature values across time from differentiation day 28–35 for each group. The MCOT is not a rate, but a coefficient that is described in detail in the methods section. Top, 72CAG vs 20CAG, middle KO vs 20CAG and bottom KO vs 72CAG. The values listed in the table denote the interaction between the time variable and the disease status variable scaled by the change over time in the reference (72 CAG or *HTT* KO versus control) and expressed as percentages. The color reflects the degree of change between the two groups and the value in the middle is the percent change and the direction. For example, the area feature in 72CAG eCNs increases by 1211 % compared to control eCNs whereas the KO is changing by 556 % but in the opposite direction. n.s. = not significant. The delta represents passage of time between the images on the left and the images on the right. (E) Illustration summarizing how each genotype shows differences in morphology and their respective rates of change with respect to time.

We next asked if CAG-expanded and *HTT* KO eCNs displayed differences in cell health compared to control eCNs. Previous reports found that CAG-expanded neurons displayed a higher rate of death than controls (HD iPSC Consortium, 2012; HD iPSC Consortium, 2017). To assess survival, we transduced eCNs with the morphology marker EGFP driven by the Synapsin promoter (Synapsin:EGFP from SignaGen) on differentiation day ~24, and imaged them daily using robotic automated longitudinal imaging (Robotic Microcopy [RM]) (Fig. 1B,C, Supplemental Fig. 4 A) to determine the cumulative risk of death of each line as previously described (HD iPSC Consortium, 2012; HD iPSC Consortium, 2017; Arrasate and Finkbeiner, 2005; Arrasate and Finkbeiner, 2012; Arrasate et al., 2004; Barmada et al., 2010; Bilican et al., 2012; Miller et al., 2011; Mitra et al., 2009b; Serio et al., 2013; Tsvetkov et al., 2013). Fluorescent eCNs displayed typical neuronal morphology with an oval-shaped cell body and numerous axonal or dendritic processes (Supplemental Fig. 4 A). We observed a large range of death rates across all lines and experiments (Supplemental Fig. 4B), therefore we used a Cox proportional hazards mixed effects model (Therneau, 2020) that accounts for batch, clone as well as unknown variability, to compare groups. This statistical approach showed that eCNs that contain 72 CAG repeats (72CAGn1/n2/n3) die significantly faster than control eCNs (20CAGn3/n4) (Hazard ratio (HR) = 2.2, $p=3.7\;{\text{E}}^{-05}$). There was no statistically significant difference between the controls and the *HTT* KO eCNs (Fig. 2A).

We next examined a second cellular hallmark, neurite length, which is correlated with neurodegeneration (Fiesel et al., 2011; Mangieri et al., 2014) and neurodevelopment (HD iPSC Consortium, 2017; Prem et al., 2020; Todd, 1992). We quantified the length of each process from each eCN soma, as previously reported (Tian et al., 2019), in images acquired from RM on differentiation day ~32. We found that CAG-expanded lines displayed subtly longer neurites than the controls (Fig. 2B). While this result appears to contradict the increased risk of death in the 72CAG eCNs, as dying neurons display contracting or blebbing neurites, and results from late stage-differentiated cortical neurons (DIV 130) from patient- derived lines (Mehta et al., 2018), it is consistent with previous observations from neurons from HD patient lines at a similar differentiation stage (HD iPSC Consortium, 2017) and other ESC-based models of HD *in vitro* (Lorincz and Zawistowski, 2009), and from neurons in the brain of early-stage HD patients (Curtis et al., 2005; Ferrante et al., 1991; Kurup and Kurup, 2003). In contrast, the *HTT* KO eCNs displayed subtle but significantly shorter neurites than controls (Fig. 2B). Thus, the differences in neurite length may represent neurodevelopmental alterations across the groups as opposed to neurodegenerative ones but could also contribute to increased vulnerability to cell death.

To capture neurodevelopmental changes, we devised a novel platform using feature extraction commonly used in ML to examine morphological changes at a single instance or across time during neuronal differentiation and maturation between the control, CAG-expanded neurons and HTT KO neurons. D24 eCNs transduced with synapsin::EGFP were imaged as described above using RM and processed in our image-processing pipeline Galaxy software (Afgan et al., 2018) (Supplemental Fig. 4C) and cropped so that only the cell body and immediate processes were captured on a cell-by-cell basis (Fig. 2C, Supplemental Fig. 4C). These cropped images were then subjected to our custom-built ML feature-based platform that is a collection of algorithms that relay information about structure on a cell-by-cell basis (Supplemental Fig. 4D).

At a single time point (differentiation day 28), we detected subtle, but highly significant and reproducible differences across a small subset of the features. For instance, the perimeter of each cell as captured by the “Edge” feature captures the perimeter of each object and relates to both size or complexity of an object (Canny, 1986). The ‘Edge” in the 72CAG eCNs is slighlty higher, suggesting a more complex shape compared to the controls (Supplemental Fig. 5A). This is consistent with the observation that the 72CAG eCNs have longer neurites than controls (Fig. 2B). Likewise, the “Edge” of *HTT* KO eCNs was smaller than controls, also consistent with observation that they have shorter processes (Fig. 2B). The “eccentricity” feature captures of the roundness of the cell (e.g. round versus elliptical) (Zhou et al., 2019) showed that the 72CAG eCNs were rounder than the controls at this time point (Supplemental Fig. 5B). We also observed that the median number of processes emanating per soma captured by the “median Sholl” feature was significantly different in the *HTT* KO eCNs compared to control and 72CAG eCNs (Supplemental Fig. 5C).

However, these differences across groups at day 28 were small. Previous studies have reported developmental alterations in HD patient derived i-neurons (HD iPSC Consortium, 2017; Conforti et al., 2020; Galgoczi et al., 2021; Mattis et al., 2015; Smith-Geater et al., 2020; Kaye et al., 2022; Ring et al., 2015), and we wondered if similar changes occurred in the eCNs. Therefore, we examined if these features changed over time to capture morphological changes that could reflect developmental shifts. eCNs that expressed synapsin::EGFP were imaged using RM as described above at differentiation day ~28 and again at day ~35 (Fig. 2C). Within each group of eCNs, all features displayed robust differences from the first time point to the last time point suggesting that the morphology of eCNs changes over time during differentiation (Supplemental Fig. 6). To estimate this shift over time, we modeled the shift across the two time-points due to a change in disease status via an interaction term (Kuznetsova et al., 2017; R Core Team, 2012) we call the “Morphology Change Over Time” or “MCOT”(the time:disease status interaction terms were scaled by the change over time in the 72CAG and *HTT* KO or control and expressed as percentages) (Fig. 2D). We evaluated features of eCNs that harbored the 72CAG or *HTT* KO compared to control eCNs. We observed that more than 1/2 of the features (11 features out of 18 total) displayed significant MCOTs in the 72CAG eCNs compared to controls, ranging from subtle (~15 %) to 12-fold (1200 %). Further, the *HTT* KO eCNs also displayed robust MCOTs of features compared to control eCNs (Supplemental Fig. 6).

The most notable features capture changes in cell size, shape, complexity and texture and are summarized in a heatmap in Fig. 2D. For example, the “cell area”, which captures the size of the cell, was found to change at a larger rate in the 72CAG eCNs compared to controls which were more static. The increase was ~1200 % greater in the 72CAG eCNS compared to controls (Fig. 2D and Supplemental Fig. 6 A). In an opposite fashion, the “cell area” decreased in the *HTTKO* compared to the 72CAG and controls (Fig. 2D and Supplemental Fig. 6 A). The change in cell size may reflect rates of cell growth that capture developmental trajectories that arise as a result of loss of HTT as opposed to those that are caused by the CAG repeat expansion.

The MCOT for “edge” was larger in the control compared to 72CAG and *HTT* KO eCNs (as observed by a – 22 % overall change), suggesting that the control eCNs are becoming more complex faster. The changes were more dysregulated in the *HTT* KO eCNs compared to the controls or 72CAG eCNs (by −128 % and – 150 % respectively) (Fig. 2D and Supplemental Fig. 6C). The “eccentricity” feature at day 28 suggested that the 72CAG eCNs were rounder than the control cells, however the MCOT for eccentricity was higher in 20CAG eCNs compared to the 72CAG eCNs (as observed by a – 66 % difference) indicating that the control eCNs became rounder faster than 72CAG eCNs during this stage of development (Fig. 2D and Supplemental Fig. 6D). The *HTT* KO MCOT for “eccentricity” was comparable to the control but was significantly different from the 72CAG eCNs, suggesting that this feature may capture a morphological change that is specific to the CAG expansion and may be interpreted as a “gain-of-function” property (Fig. 2D and Supplemental Fig. 6D).

Other features capture information about an objects’ texture such as the Gray-Level Co-occurrence Matrix (GLCM) (Iqbal et al., 2021), which measures the spatial relationships between neighboring pixel intensities. We observed that the “GLCM energy” feature (Sullivan and Feinn, 2012) increased in all lines over in time in culture, suggesting that that the eCNs smoothen and become less textured as they differentiate. The MCOT of the 72CAG eCNs differed from controls by ~27 % showing less change over time (Fig. 2D and Supplemental Fig. 6F). There was no difference between the 72CAG compared to the *HTT* KO eCNs, suggesting that this feature may be capturing a cellular rate of change that reflects a loss of function due the CAG expansion. MCOTs for other GLCM-like features, such as GLCM Dissimilarity (which measures contrast between pixels) and GLCM Corr (measures the linear relationship between pixel pairs) (Iqbal et al., 2021; Sullivan and Feinn, 2012), were only significantly different in the *HTT* KO eCNs (Fig. 2D and Supplemental Figs. 6G,H). This suggests that these features capture a true loss of HTT function that is distinctly different from the loss of function that occurs from the CAG repeat expansion.

Additional features capture information about an objects’ complexity such as “HOG” (histogram of gradients) (Li et al., 2016) or Minkowski-Bouligand Fractal Dimension (Mink Dim) (Squarcina et al., 2015), which has been used to detect differences in complexity in human tissues (Chappard et al., 2001; Squarcina et al., 2015), and the “sobel noise” which is an edge detector based on gradient changes across an image (Jia et al., 2014). The sholl intersection (Kutzing et al., 2010) feature captures the number of processes protruding from a soma, which can indicate the pattern of neurite outgrowth. The majority of “complexity” features decreased over time all groups, suggesting a decrease in cell-shape complexity as the neurons matured. However, the MCOT for complexity features decreased more in the control eCNs compared to the 72CAG or *HTT* KO eCNs (Fig. 2D and Supplemental Fig. 6 I, J, K, L). There are other features that display differences across the control, 72CAG and *HTT* KO eCNs (Supplemental Fig. 6B, E, M, N, O,P Q), but the biological meaning of these features is unclear.

To summarize, 72CAG eCNs grow larger faster than control eCNs, which are more static, whereas *HTT* KO eCNs grow more slowly and stay smaller than both the 72CAG and control eCNs (Fig. 2D, E). The texture and complexity of the neurons increases most robustly in the control eCNs compared to the 72CAG and *HTT* KO eCNs (Fig. 2D, E). These results suggest that *HTT* CAG repeat length expansion and loss of HTT function both influence cortical neuron morphology and development, however the CAG repeat influences metrics in ways that cannot be explained by HTT loss of function alone.

### 2.3. Overview of OMIC analysis of ESCs and eCNs

To explore the gene expression, protein and epigenetic profiles of the isogenic cell lines, we conducted transcriptomics (RNAseq), proteomics (mass spectrometry), and epigenomics (ATACseq and ChIPseq with H3K4me1, H3K4me3, H3K27ac and H3K27me3 histone marks) at both the pluripotent and eCN stage as described in Methods (Neuro et al., 2021; Demichev et al., 2020; O’Neill and Turner, 2003; Thorne et al., 2004). Two control cell lines (20CAGn1/n2), two expanded repeat lines (56CAG and 72CAGn1) and the *HTT* KO cell lines were collected across 3 successive passage replicates at the pluripotent stage to establish a baseline cell signature. Similarly, eCNs were generated in three sets of independent differentiations. Generated eCN’s were assessed to by ICC and in general, these cultures contain a high percentage of MAP2-positive signaling area as well as neurons that express forebrain markers FOXG1 and TBR1, with some NESTIN-positive progenitors remaining (Supplemental Fig. 7 A). While eCN populations from all cell lines did express the expected markers, there was some variation from the imaging studies and between the different genotypes (Supplemental Fig. 7). The only significant differences found between genotypes were for FOXG1 expression with cultures generally expressing ~45 % positive nuclei, however KO and CAG expanded eCNs contain higher expression than that of the controls ($\beta =0.795,p<5.97\times 10^{-5}$ and $\beta =1.28,p<5.99\times 10^{-6}$ respectively, Supplemental Fig. 7B). TBR1 is expressed in ~65 % of the eCNs with no significant difference in expression between genotypes (Supplemental Fig. 7C), while BCL11B is expressed at ~20 % (Supplemental Fig. 7D) with no significant difference in expression between genotypes. Pooled pellets from each line at the pluripotent and eCN stage were subjected to the same set of assays. All replicates for both pluripotent and eCN RNAseq samples were included after meeting ENCODE3 quality benchmarks with greater than 50 million reads (Supplemental tables 4,5). All proteomics samples showed comparable metrics for sample preparation (protein count) and data acquisition (total ion current, mass peak intensity, spectral ratio) and were each included as well (Supplemental tables 4,5). For the epigenomics samples, we included data from replicates that met ENCODE3 benchmarks for minimum quality, which included the majority of cell lines (Supplemental tables 4,5). Finally, we integrated the primary assay data using the OmicsIntegrator tool (Fig. 1C), using the differential analyses as inputs to the Prize-Collecting Steiner Forest algorithm (Neuro et al., 2021; Tuncbag et al., 2016). The algorithm identifies the highest-confidence protein-protein interactions involving differential omics from a reference of known protein-protein interactions.

### 2.4. HTT KO has the strongest omics effect in pluripotent cells, and eCN stage cells display clustering based on CAG length and HTT KO

Each of the pluripotent omic data sets (RNAseq, ATACseq, ChIPseq, and DIA-mass spectrometry) were subjected to unbiased clustering analysis prior to differential analyses. Principal component analysis (PCA) for transcriptional data showed that all growth replicates of each cell line clustered closely (Supplemental Fig. 8 A); however, the lines did not cluster by genotype (CAG expanded vs control). The 20CAGn1 and 20CAGn2 lines were distinct from the 56CAG and *HTT* KO cells along principal component 1 (Supplemental Fig. 8 A) and from 72CAGn1 cell lines along principal component 2 (Supplemental Fig. 8 A). The separation of controls from *HTT* KO or CAG repeat length-expanded cells suggests transcriptional differences even at the iPSC stage. The two CAG-expanded lines (56CAG and 72CAGn1) did not cluster together (Supplemental Fig. 8 A), reflecting some differences between the two repeat lengths. We also observed that 56CAG and *HTT* KO cells clustered with each other on the first principal component (Supplemental Fig. 8 A). This observation could suggest that at pre-developmental stages, adult-onset HD-associated CAG repeat expansion (56CAG) were more closely associated with *HTT* loss of function.

We investigated whether the similarities and differences between genotypes inferred from PCA were reflected in the number of differentially altered signals. Despite their clustering together in the PCA, we treated 56CAG and *HTT* KO as separate populations to learn more about what transcriptional changes were shared or unique when compared to controls. The most highly expanded line, the 72CAGn1 line, had fewer differential transcriptomic changes and differential ATAC-seq peaks compared to the controls (20CAGn1 and 20CAGn2) relative to the number of differentially expressed genes and differentially accessible peaks between *HTT* KO cells and controls as well as the 56CAG cells and controls (Supplemental Fig. 8B, Supplemental Table 6, 7). Transcriptomic data validated the decrease in HTT expression (Supplemental Fig. 8C). Data integration analysis using the Omics Integrator tool showed one cluster of particular interest associated with *HTT* KO (Supplemental Fig. 8D). In this cluster, the HTT protein is a node connected to its known interactor CREBBP/CBP (Steffan et al., 2001) as well as its downstream target, FOS. The HTT protein also interacted with TBP, which acts in a complex with CREBBP and HTT for transcription-coupled repair (Gao et al., 2019). Additional nodes TOP2A and FOXM1 potentially highlight HTT’s normal role in DNA replication and repair.

**Fig. 3.**
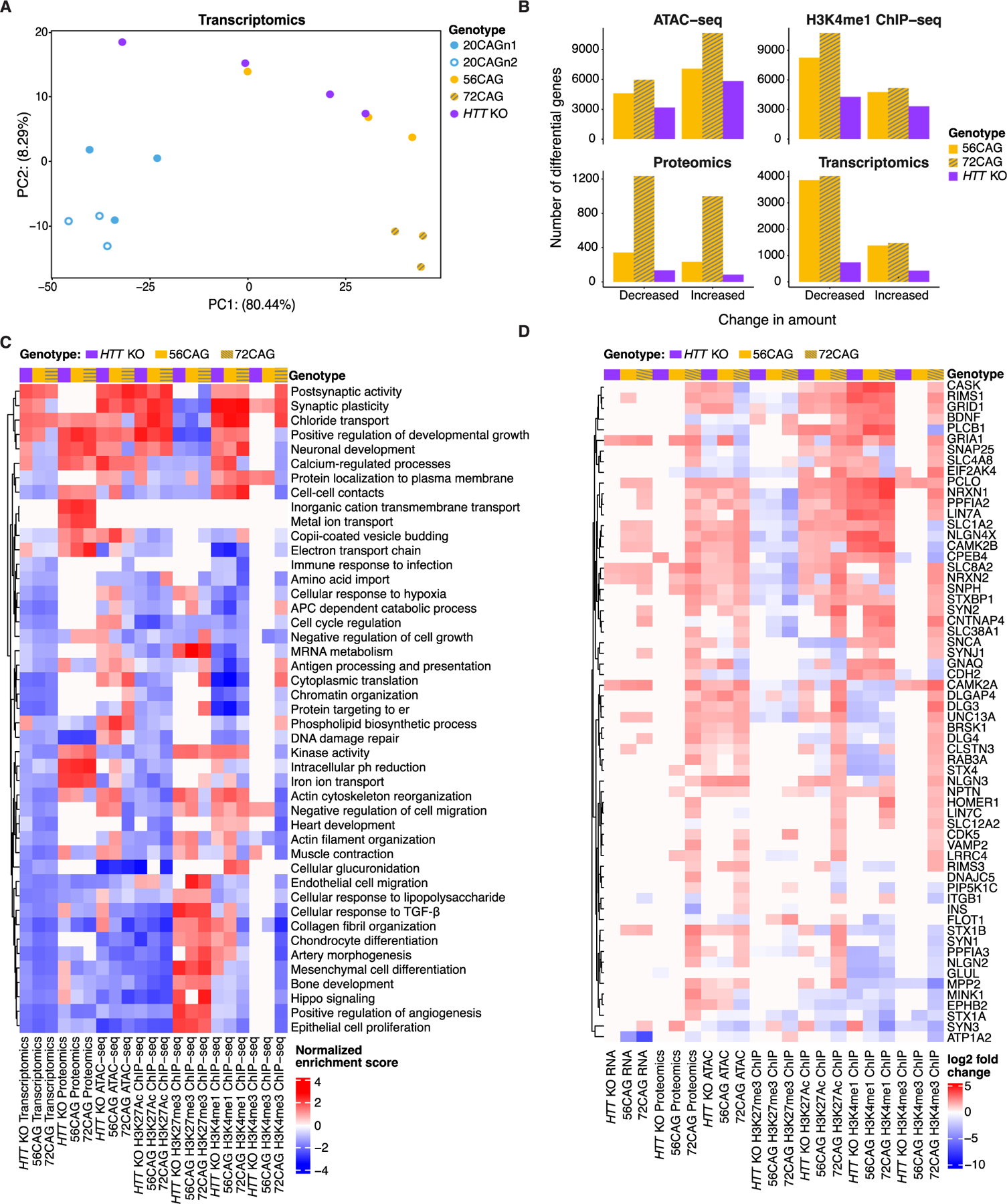
Analysis of primary assay data shows similarities across data types and between HD cells and *HTT* knockout cells. (A) PCA of cortical neuron transcriptomics shows partial separation by CAG expanded status along principal component 1. (B) Number of differential genes in ATAC-seq, H3K4me1 ChIP-seq, proteomic and transcriptomic data comparing 56CAG eCNs to control neurons (Gold bar), 72CAG eCNs (Gold bar), and *HTT* KO to control neurons (Purple bar) (C) Heatmap of the normalized enrichment scores of the union of significantly enriched pathways in transcriptomic, epigenomic and proteomic data in 52CAG eCNs, 72CAG eCNs, and *HTT* KO eCNs. Many pathways have the same direction of change in all three genotypes in each assay. Moreover, there exist pathways that are upregulated in all assays but downregulated in H3K27me3 ChIP-seq, which is a repressive epigenetic mark. (D) Heatmap of the log_2_ fold changes of the union of differentially expressed genes across assays and genotypes that are involved with postsynaptic activity. The genes that are displayed in this pathway were differentially expressed genes in at least one comparison of genotypes and assays. Many of these genes show the same direction in all omics except the repressive histone mark H3K27me3 ChIP-seq, which changes in the opposite direction.

Alterations in expression and epigenetics were next examined at the cortical neuron stage, including HTT RNA levels (Supplemental Fig. 9 A). We utilized PCA to explore the clustering patterns of gene expression of the differentiated eCNs using the OMIC data. Both CAG expansion and loss of HTT function displayed significant effects on gene expression, protein levels and epigenetic readouts in the eCN population. CAG repeat expansion and HTT loss accounted for the majority of variance by PCA, however some variance was observed between replicates, likely due to heterogeneity across independent differentiations. Control eCNs clustered across all data sets and all cell lines separated by genotype, particularly for the RNAseq, ATACseq, and H3K4me1 ChIPseq data sets (Supplemental Fig. 9B–E). In the RNAseq data, CAG repeat seemed to account for the variability across PC1, with the 72CAG line clustering at one end and the control lines clustering at the other (Fig. 3A). However, since the first principal component for the transcriptomics PCA accounted for such a large percentage of the variance (80 %), we used Gene Ontology (GO) to analyze the top 50 most variable genes (Supplemental Table 8, 9). We saw enrichment in processes associated with development and differentiation; however, none were specific to either neural or neuronal cell types and none were associated with either mitochondrial or ribosomal genes, suggesting the variance was not due to RNA composition or cell type differences. For the ATAC-seq peaks and protein expression in eCN cell lines, we observed separation by the CAG expansion for both 56CAG and 72CAGn1 along PC1, while in the H3K4me1 peaks, we observed this separation along PC2 (Supplemental Fig. 9C–E). In the PCAs, *HTT* KO cells tended to cluster with the CAG-expanded repeat cell lines, suggesting some common changes. We also performed PCA on other ChIP-seq data sets, however, the other epigenomic marks did not show separation by repeat length (Supplemental Fig. 9F–G).

### 2.5. Pathway analysis and multi-omic differential analyses suggest coordinated changes across HTT genotypes

In eCNs, we observed 4084 differentially expressed genes (DEGs) and 5506 DEGs of the 56CAG and 72CAGn1 compared to controls, respectively (Fig. 3B, Supplemental Table 10, 11, FDR < 0.01, logFC>|1|). The number of DEGs for the *HTT* KO eCNs compared to controls were far fewer at 1168 (Fig. 3B, Supplemental Table 10, 11 FDR < 0.01, logFC>|1|). Many of the DEGs in CAG-expanded eCNs overlapped with those identified in previous studies comparing HD to control iPSC-derived cortical (Mehta et al., 2018) and striatal neuron (HD iPSC Consortium, 2017) populations (Supplemental Fig. 10 A,B, hypergeometric p-value = 2.0*10^−6^ and hypergeometric p-value<10^−16^ respectively). There was also significant overlap between the DEGs we identified in *HTT* KO eCNs compared to published results in iPSC-derived striatal neurons (HD iPSC Consortium, 2017) (Supplemental Fig. 10C, hypergeometric $p=3.8^{*}10^{-4}$) and partial overlap with published results in cortical neurons (Supplemental Fig. 10D, hypergeometric p=0.02). The overlap between DEGs identified in RUES2 lines and those found in iPSC-derived neurons from HD patients supports the utility of the isogenic eCN-HD model and its ability to discriminate between loss-of-function versus gain-of-function cellular changes attributable to the CAG expansion.

Similar to the transcriptomic data, we identified 573 and 2234 differentially expressed proteins (p<0.05, logFC>|0.6|) in the 56CAG and 72CAG repeat lines compared to control eCNs, but only 217 differentially expressed proteins comparing *HTT* KO to control eCNs (Fig. 3B). The ATAC-seq and ChIP-seq data also had a greater number of differential peaks for the CAG expansion comparisons (Fig. 3B, Supplemental Table 10, 11). Specifically, we found 11,676 and 16,587 differential ATAC-seq peaks for the 56CAG and 72CAGn1 repeat lines compared to controls (Fig. 3B, Supplemental Table 10, 11).

Pathway analysis for each of these comparisons was performed across all data sets by gene set enrichment analysis (GSEA). Not surprisingly, considering HTT’s putative role in neurodevelopment (Liu and Zeitlin, 2017; McKinstry et al., 2014; Dragatsis et al., 2000) and the early stage of the cortical neurons analyzed, several pathways relating to development and proliferation were enriched across multiple genotypes and assays (Fig. 3C). These included Hippo signaling, developmental growth, and cell cycle regulation. For example, developmental growth regulation was enriched in *HTT* KO, 56CAG and 72CAG proteomics, *HTT* KO, 56CAG and 72CAG H3K27me3 ChIP-seq, and *HTT* KO and 72CAG H3K27Ac ChIP-seq. Other pathways of interest included DNA damage repair, synaptic plasticity, chromatin organization, and negative regulation of cell migration (Fig. 3C). The implicated DNA damage repair genes included important enzymes such as PARP1, XRCC1 and OGG1 as well as replicative enzymes like PCNA, POLE and POLD1, the latter identified as a candidate modifier of age-of-onset in HD (Lee et al., 2022).

Some pathways had related enrichment scores across *HTT* genotypes. For example, some pathways that were significantly upregulated in *HTT* KO eCN transcriptomic data were also upregulated in the CAG-expanded eCNs by transcriptomic analysis (Fig. 3C). Some of these enrichments in the transcriptional data were mirrored by significant enrichments in proteomics, ATAC-seq or activating ChIP-seq marks, and significant down-regulation of the repressive H3K27me3 mark (Fig. 3C). A significant proportion of genes that were differentially altered in each assay had the same direction of fold change in *HTT* KO and CAG-expanded eCNs, designated as “concordant changes” (Supplemental Fig. 11 A FDR-adjusted hypergeometric test p-value less than 0.1). As an example, genes involved in postsynaptic activity showed coordinated changes across multiple genotypes (Fig. 3D). Several genes had concordant changes across genotypes in more than one assay (Supplemental Fig. 11B, C). The presence of these concordant changes across *HTT* genotypes and assays supports the hypothesis that some of the pathways are similarly regulated in *HTT* KO and CAG-expanded repeat neurons, and that CAG repeat length expansion may at least in part cause reduced normal function of HTT.

### 2.6. Network analysis shows similarities between biological processes in CAG- expanded and HTT KO eCNs

**Fig. 4.**
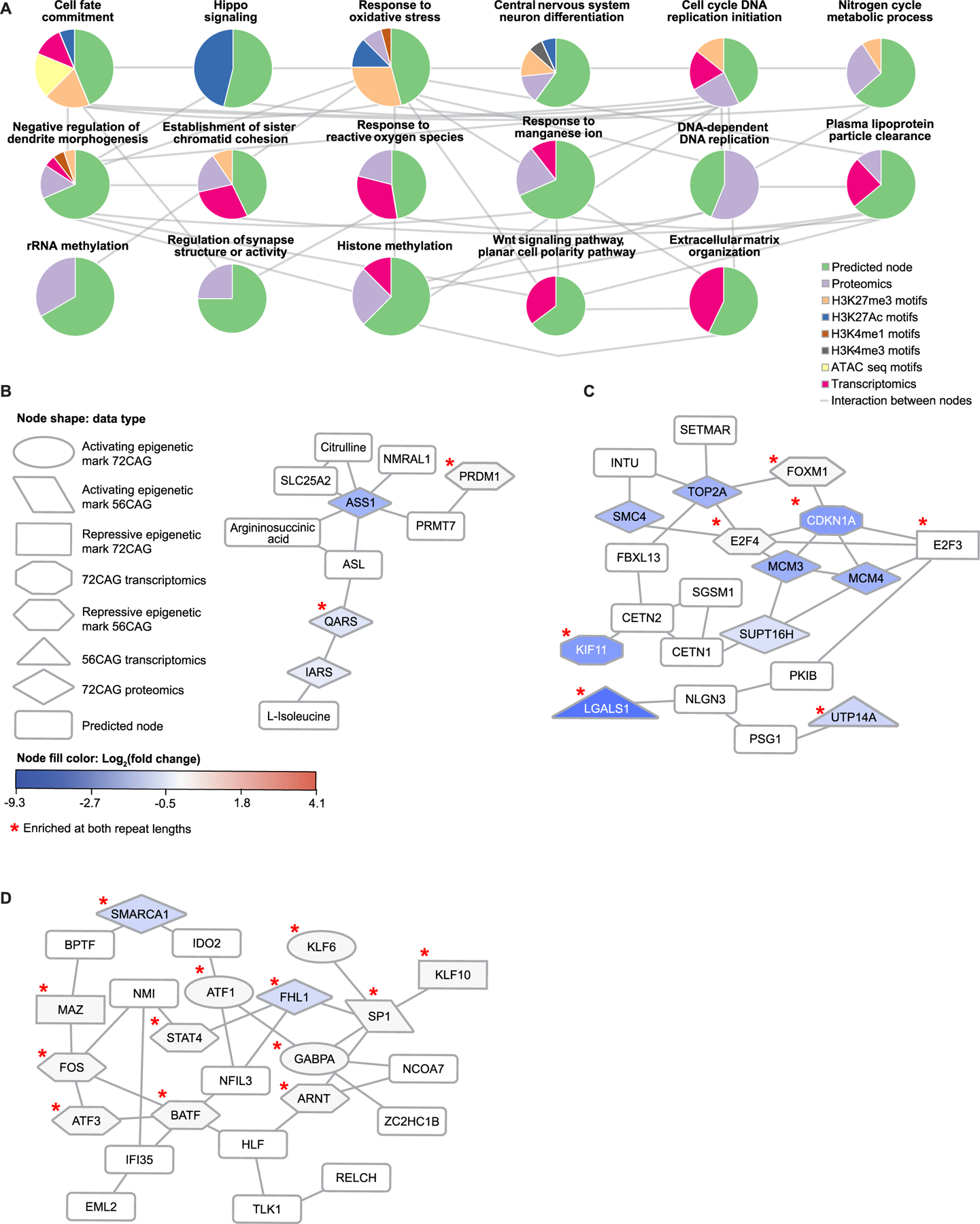
Mutli-omic network integration of CAG expansion-associated -Omics. (A) Multi-omic integration results for proteomics, epigenetics and transcriptomics enriched in 56CAG and 72CAG eCNs compared to controls. Each pie chart represents the origin of the nodes in the subclusters and is labeled with the enriched GO Biological Process. Edges between the pie charts indicate whether at least one node in one subnetwork interacts with at least one other node in the other subnetwork. (B) Subnetwork enriched for nitrogen cycle metabolic processes. Red asterisks indicate nodes that are enriched in the 72CAG and 56CAG eCNs. (C) Subnetwork enriched for DNA-dependent DNA replication processes includes nodes that are necessary for the DNA damage response process. Red asterisks indicate nodes that are enriched in 72CAG and 56CAG eCNs. D) Subnetwork enriched for response to reactive oxygen species identifies proteomic, epigenomic and predicted interactors that are involved in the response to reactive oxygen species. Red asterisks indicate nodes that are enriched in 72CAG and 56CAG eCNs.

We used the differential signals between CAG-expanded eCNs vs. control eCNs to identify disease-relevant subnetworks. Specifically, we used the results of differential analyses comparing 56CAG to control and 72CAG eCNs to control as inputs to OMICS integrator (Tuncbag et al., 2016) which, as described above, identifies the highest-confidence protein-protein interactions from a reference of known protein-protein interactions. We refer to nodes that were not identified in our set of differential omics but were included in the network solution as “predicted nodes”. Louvain clustering of our pruned protein-protein interaction network showed subnetworks comprised of proteins that participate in core cell signaling pathways such as cell fate commitment, Hippo signaling, response to oxidative stress, histone methylation, extracellular matrix, cell cycling, and WNT signaling among others (Fig. 4A). Many of these processes have been previously associated with HD. Support for these enriched pathways comes from multiple lines of evidence across omics modalities (Figs. 4B–D). Examples of subnetworks enriched for HD-associated processes that are supported by interactions among enriched proteins, epigenetic motifs and differentially expressed genes include those enriched for response to reactive oxygen species, nitrogen cycle metabolic processes and DNA-initiated DNA replication. In these subnetworks, at least half of the differential signals were seen in comparisons between 56CAG cortical neurons vs. control as well as 72CAG cortical neurons vs. control (Fig. 4B–D). The subnetwork enriched for DNA-initiated DNA replication shared interactions among many of the same MCM proteins observed in iPSC-derived Huntington’s disease medium spiny neurons in previous studies (Fig. 4C (Tshilenge et al., 2023),). These results suggest that increased *HTT* repeat length is associated with reduced levels and potentially reduced activity of proteins involved in biological processes previously associated with HD (Fig. 4).

**Fig. 5.**
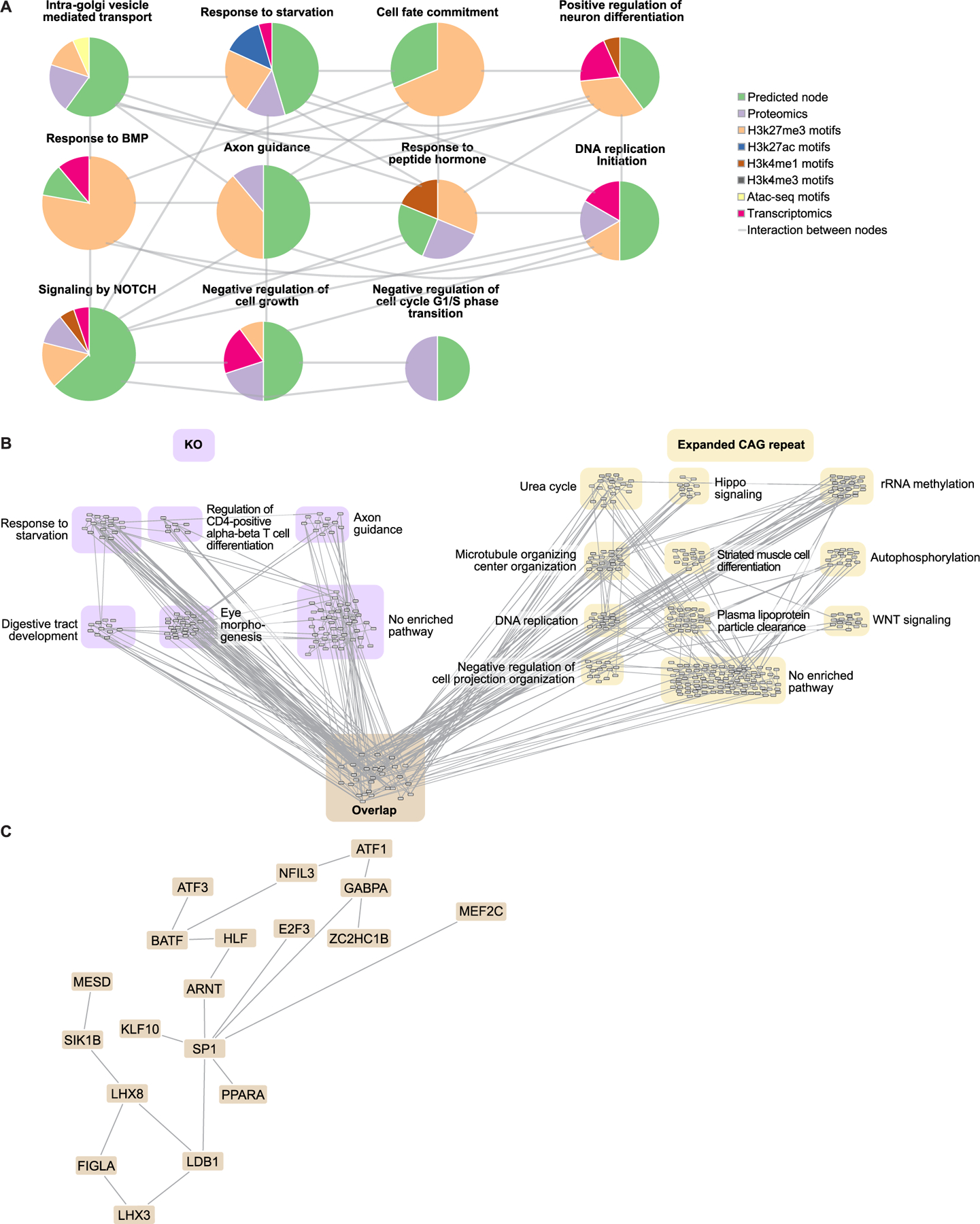
Integrative network analysis of *HTT* KO multi-omics show distinct and shared nodes and biological processes to the CAG expansion network. (A) Louvain clusters of the integrative network of differential proteomics, epigenomics and transcriptomics between *HTT* KO and control. The subnetworks are indicated by a pie chart and are labeled by the enriched GO Biological Process. Pie charts indicate the proportion of nodes represented by a given molecular data type. Edges between the pie charts indicate whether at least one node in one subnetwork interacts with at least one other node in the other subnetwork. (B) The union of nodes in the *HTT* KO and expanded CAG networks show that there exists a significant overlap in the nodes in the *HTT* KO and expanded CAG networks. The union of nodes between the two networks were re-clustered and labeled with enriched biological processes, with the KO nodes highlighted in purple, the HD nodes highlighted in gold, and the overlapping nodes highlighted in orange. The overlapped nodes are enriched for targets of MAPK signaling. (C) The largest connected component of the nodes and edges that are at the intersection of the *HTT* KO and HD networks.

We also performed network-based multi-omic integration for data from *HTT* KO eCNs, revealing that some subnetworks were enriched for processes relevant to neuronal development, including axon guidance, NOTCH signaling, cell fate commitment and regulation of cell growth (Fig. 5A).

Some of the processes enriched in the *HTT* KO network were also enriched in the CAG-expanded network, including neuronal differentiation, cell fate commitment and DNA replication initiation. To understand the degree of overlap, we visualized the union of the two networks (Fig. 5B), clustering the unique and overlapping components separately. There were 29 nodes shared between the *HTT* KO and CAG-expanded repeat networks, which is greater than the number expected by chance between two networks of similar size (Fig. 5C, Supplemental Fig. 12 A, Supplemental Table 12). The overlapped network also had a larger connected component than any intersection of two random networks of the same size, supporting the hypothesis that the intersection of these two networks represents biological pathways as opposed to disconnected but shared nodes (Supplemental Fig. 12B). Among the nodes that overlapped are MEF2C, ATF1, and MEF2A, which are targets of MAPK signaling (Gupta and Prywes, 2002; Zhao et al., 1999). Neuronal development-associated nodes were also found in this intersection such as SP1 and LHX3. The overlapped network highlights possible biological processes that are affected due to a loss of HTT function that occurs either as a result of a total loss of the protein or of the CAG expansion (Supplemental Fig. 12C).

The networks also revealed pathways specific to each genotype. *HTT* KO-specific nodes were enriched for starvation response and axon guidance, while the CAG-expanded exclusive nodes were enriched for Hippo signaling and WNT signaling as well as metabolic processes such as the urea cycle and plasma lipoprotein particle clearance (Fig. 5B). These distinct networks may reveal pathways that are unique to the gain of functional activity stemming from the expanded CAG repeat versus.

**Fig. 6.**
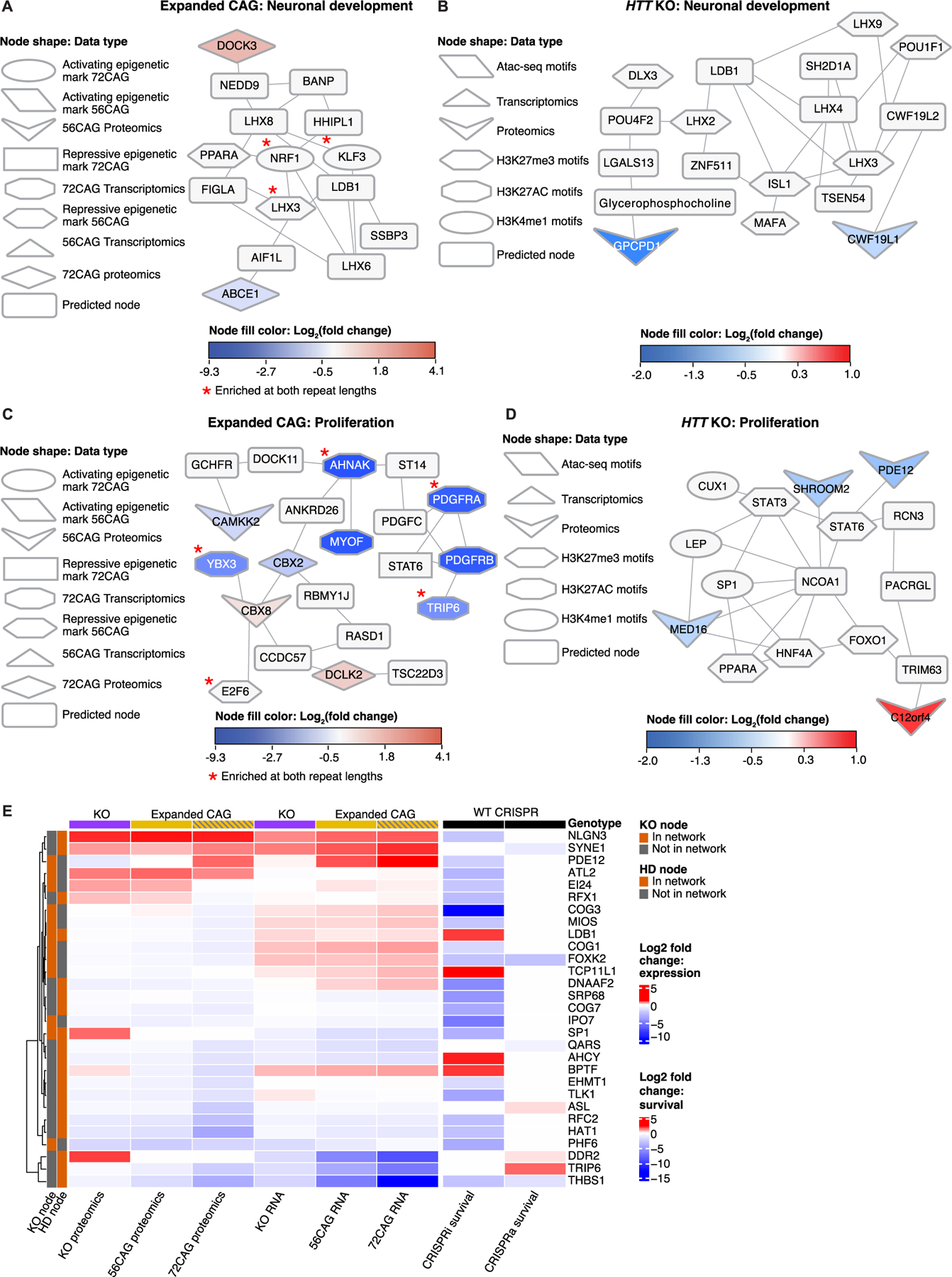
Subnetworks in the expanded CAG repeat and *HTT* KO networks show interactions that implicate similar biological processes despite differing node identities. (A) an expanded CAG subnetwork shows interactions between LHX3, LHX6, and LDB1, which are nodes associated with neuronal development. (B) An *HTT* KO subnetwork shows interactions between LHX3, ISL1, LHX4, and LDB1, which are nodes associated with neuronal development. (C) An expanded CAG subnetwork shows interactions between CBX8 and E2F6, which are nodes associated with cellular proliferation. (D) In an *HTT*KO subnetwork, we observe associations between MED16, HNF4A and FOXO1. HNF4A and FOXO1 are nodes associated with proliferation. Red asterisks in (A) and (C) indicate nodes that are enriched in 72CAG and 56CAG eCNs. (E) Heatmap showing the changes in the multi-omic data for nodes that appear in the *HTT*KO network or the CAG expanded network and significantly influence glutamatergic neuronal viability in a CRISPRi or CRISPRa viability in previously published data (Tian et al., 2021).

Interestingly, in some cases, the CAG-expanded and *HTT* KO networks reflected similar processes, even when there were few overlapping nodes. Both networks showed an enriched H3K27me3 motif for the DNA-binding protein LHX3. We found that the LHX3 protein interacted with other nodes involved in neuronal development (Fig. 6A, B). LHX3 is a homeodomain protein essential for nervous system development including of motor neurons, interneurons and pituitary development (Thaler et al., 2002). In the CAG-expanded networks, LHX3 interacted with the predicted nodes containing LHX6 and LDB1 that are involved in neuronal development (Fig. 6A). Similarly, in the *HTT* KO network, LHX3 interacted with LHX4, LDB1 and ISL1, which are involved in neuronal differentiation (Bhati et al., 2017) (Fig. 6B). Like LHX3, the ISL1 motif was enriched in the H3K27me3 ChIP-seq data. H3K27me3 is a repressive epigenetic mark, suggesting that both LHX3 and ISL1 are associated with reduced gene expression activity in *HTT* KO (Fig. 6B). In the KO network, LHX3 was in the same subnetwork as CWF19L1, which is a gene commonly associated with spinocerebellar ataxia (Fig. 6B).

We also observed a subnetwork in the CAG-expanded network that showed interaction between CBX8, a transcriptional repressor, and E2F6, a proliferation-associated transcription factor (Fig. 6C). Since CBX8 is upregulated in the CAG-expanded eCNS as seen by proteomics and E2F6 is a motif associated with the repressive H3K27me3 mark, this subnetwork suggests associations with reduced proliferation and increased CBX8-mediated transcriptional repression. Similarly, we observed associations between MED16 and proliferation-associated transcription factors such as HNF4A and FOXO1 in the *HTT* KO network (Fig. 6D). MED16 part of the mediator complex, which coactivates RNA polymerase II and promotes transcriptional activation (Myer and Young, 1998; Kim et al., 2004), and the proliferation-associated epigenetic motifs were derived from H3K27me3 ChIP-seq data (Fig. 6D). MED16 was downregulated in the HTT KO proteomics data (Fig. 6D). These results suggest that, despite involving different nodes, the *HTT* KO network and CAG-expanded network represent protein-protein interactions involving similar changes in the regulation of transcription and proliferation (Fig. 6C, D).

To understand how the nodes identified in our network analysis might affect neurodegeneration, we next analyzed our omics data and previously published perturb-seq data that measured single-cell differential transcriptomic changes in iPSC-derived glutamatergic neurons after CRISPRi knockdown or CRISPRa activation of target genes (Tian et al., 2021) (Fig. 6E). Specifically, after identifying genes common between the perturb-seq study and our own network analysis, we assessed whether knockdown or activation of these genes was associated with altered neuronal survival. Indeed, genes that were down-regulated in CAG-expanded and in *HTT* KO eCNs, such as *RFC2*, *THBS1*, *HAT1*, *PHF6*, *IPO7*, and *COG7*, were previously found to be associated with reduced neuronal survival upon knockdown, suggesting that these genes may have a role in HD-associated neurodegeneration (Fig. 6E).

Our analysis identified a few possible, if challenging, targets for therapeutic intervention. Knockdown of *BPTF, AHCY, TCP11L1*, or *LDB1* improves glutamatergic neuronal survival (Fig. 6E). *BPTF, TCP11L1* and *LDB1* were also upregulated in the CAG- expanded and *HTT* KO transcriptomics (Fig. 6E). However, at the proteomic level, BPTF, TCP11L1 LDB1 were downregulated except for BPTF in *HTT* KO, which may reflect changes to protein homeostasis or compensatory effects (Fig. 6E). Differences in transcriptomics and proteomics can be a consequence of compensatory effects and feedback loops, or differences in degradation pathways among other reasons. It will be of interest to determine the effects of genetic perturbation of *BPTF, TCP11L1* and *LDB1* on HD and assess whether these interventions would be useful therapeutic approaches, thus reconciling differences in transcriptomic and proteomic data. Additionally, the integration of RM with omics analysis could help provide more functional information by examining how perturbations affect noted differences in cellular survival, size, and eccentricity as well as changes in cell size and cellular complexity over time.

## 3. Discussion

In this study, we used a systems approach including live cell imaging and multi-omics to interrogate epigenetic, transcriptomic, and proteomic changes associated with CAG expansion and loss of HTT using an ESC-derived isogenic series of *HTT* modified lines patterned into cortical forebrain-like neurons (Fig. 1). CAG- expanded and *HTT* KO eCNs differentiated into cultures containing high levels of TBR1 and FOXG1-expressing neurons with a small subset of inhibitory neurons (Fig. 1, Supplementary Figs. 2,3). Using RM, we show that CAG-expanded eCNs displayed a neurodegenerative phenotype of dying faster over time compared to control and KO *HTT* eCNs, which is consistent with previous reports examining patient–derived HD i-neurons compared to controls (HD iPSC Consortium, 2012; HD iPSC Consortium, 2017). Previous studies investigating development in animals with homozygous deletion of the murine Huntingtin gene (*Hdh*) displayed organismal cell death as early as embryonic day ~8 (Nasir et al., 1995; Duyao et al., 1995; Zeitlin et al., 1995). Significant defects in embryonic stem cell development were also observed, with impaired neuroepithelial rosette formation observed as early as days ~5–7 *in vitro* (Lo Sardo et al., *2012*). In contrast, we do not observe increased cell death in the KO *HTT* eCNs compared to controls. Consistent with this outcome, prior studies on these same lines that lack HTT do not show pronounced phenotypic differences until later stages of neuronal development (Ruzo et al., 2018). This discrepancy may indicate that the murine homolog, Hdh, plays developmental roles distinct from those of human HTT as previously reported (Ruzo et al., 2018) or that the stage of development at which HTT is knocked out also impacts its consequence.

Our multi-omic integration analysis identified overlapping changes across genotypes as well as subnetworks unique to each genotype, highlighting changes in developmental and other pathways associated with CAG expansion and loss of HTT function. Furthermore, the robust gene expression changes across groups manifest as morphological changes such as alterations in neurite length as well as robust dynamic cell-structure changes across time. We found subtle, but significant differences with longer neurite length in CAG-expanded eCNs, consistent with previous reports, whereas neurite length in *HTT* KO eCNs was reduced in a distinctly opposite fashion (Fig. 2). Our results uncover key molecular targets and pathways for subsequent perturbation studies at the molecular and cellular level.

**Fig. 7.**
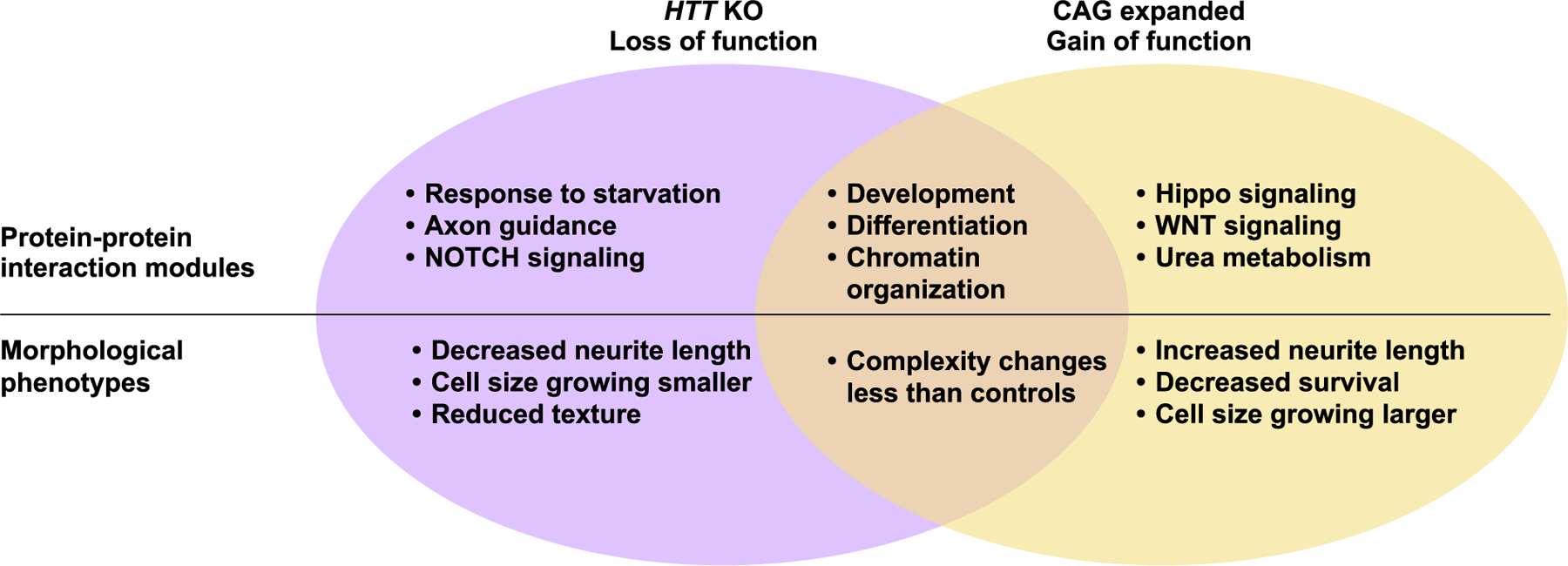
Schematic illustration summarizing overlap and unique features of CAG repeat expansion and KO.

Integration of epigenomics, proteomics and transcriptomics data from iPSC-derived models of human disease has emerged as a powerful approach for mechanistic studies of complex diseases (Neuro et al., 2021; Wang et al., 2018; Sarkar et al., 2020; Switonska-Kurkowska et al., 2021; Ooi et al., 2019), particularly for neurodegenerative diseases that display dysregulation of multiple biological processes/pathways. We used a network-based analytical framework to understand the biological processes underlying *HTT* CAG repeat length expansion and HTT knockout in an isogenic series to avoid patient-to-patient variability. By comparing the genotype-associated networks, we could infer which molecular changes were unique to CAG expansion and which had the same effect as HTT knockout, which could be especially useful for testing targets for therapeutic intervention given that current interventions are focused on total or allele-specific HTT lowering. The CAG-expanded and HTT knockout network had subnetworks enriched for similar biological processes such as cellular proliferation and neuronal differentiation (Figs. 4, 5, 6). Morphological analysis also showed changes in the rates of cortical neuron development in CAG-expanded and HTT knockout neurons (Fig. 7). We note that the common pathway enrichments between the CAG-expanded and HTT knockout networks were comprised of different nodes (Fig. 5, 6). The overlap between the two networks included MEF2C, ATF1 and MEF2A, which are targets of MAPK signaling (Fig. 5B). Neither MAPK signaling nor the set of MAPK targets were enriched in the single-omic analyses, highlighting the power of the network analysis to identify new nodes and biological processes that represent shared processes between expanded CAG repeats and HTT KO.

Early HD studies supported the idea that the CAG expansion led to gain-of-function characteristics that were toxic to cells. This included human genetic studies that observed that individuals with *HTT* deletion syndromes did not phenocopy the symptoms associated with typical HD (e.g Battaglia et al., 2015). This was also supported by early mouse models where homozygous knockout of mouse huntingtin (*Hdh*) was embryonic lethal (Duyao et al., 1995; Zeitlin et al., 1995), whereas heterozygous knockout mice survived to adulthood and were phenotypically normal (Duyao et al., 1995). Further, mutant HTT delivery is sufficient to rescue the KO mouse phenotype (White et al., 1997). However, subsequent mouse models using conditional KO as well as cell culture models have led to a reevaluation of how reduced HTT function and timing of that reduction may contribute to disease progression (Liu and Zeitlin, 2017). Brain-specific inactivation of Hdh in mice results in motor deficits and striatal degeneration, similar to that seen in mouse models of HD (Dragatsis et al., 2000), as well as cellular hallmarks of impaired autophagy (Ochaba et al., 2014). iPSC and ES-derived cell models expressing expanded CAG have impairments in processes associated with normal HTT function such as mitotic spindle orientation and transcription of BDNF (Ruzo et al., 2018; Ring et al., 2015; Godin et al., 2010; Zuccato et al., 2001). Additionally, conditional silencing of Hdh in the mouse cortex affects excitatory synapse formation resulting in an excess of hyperactive excitatory synapses in the cortex early in development (p21) that later deteriorates by 5 weeks (McKinstry et al., 2014). This same phenotype was found in the zQ175 mouse model of HD (McKinstry et al., 2014). Interestingly, synaptic function was significantly dysregulated in our data sets, with several genes such as *GRIA1*, *CASK*, and *SCNA* being upregulated in either the transcriptome, proteome, or epigenome in both CAG-expanded and HTT KO lines, suggesting that our data set likely reflects this early timepoint of dysregulation that leads to later progression of disease (Fig. 3).

Our network-based analysis identified developmentally relevant transcriptional factors SP1 and LHX3 in the overlapped subnetwork, further emphasizing neuronal development as an important process associated with HTT knockout and *HTT* CAG repeat length expansion. This observation is further supported by our analysis that there are dynamic morphological changes in eCNs (Fig. 2). SP1 as a node in this shared network is not surprising, as several studies have evaluated not only its interaction with HTT (Dunah et al., 2002) but also its potential role in HD progression (Dunah et al., 2002; Ravache et al., 2010; Qiu et al., 2006).

Previous studies using pluripotent cells showed dysregulation in pathways associated with axonal guidance (Nasir et al., 1995; Uppala and Sahr, 1997), TGFb (van der Walt et al., 2014), WNT, and BDNF signaling (Uppala and Sahr, 1997; Virtanen et al., 2020; McQuin et al., 2018) and neurogenesis particularly via NEUROD1 (Nasir et al., 1995; Virtanen et al., 2020). While correction of CAG expansion was able to rescue some dysregulation, including TGFb signaling (van der Walt et al., 2014), HTT’s known role in many of these pathways including BDNF signaling (Gauthier et al., 2004) and neurogenesis (McKinstry et al., 2014; Trier et al., 1996) suggest that HTT loss of function is contributing to these differences. Our expanded CAG transcriptional data shared significant overlap of DEGs with previous studies of patient-derived iPSC cortical (Mehta et al., 2018) and neural cells (HD iPSC Consortium, 2017). Overlapping genes with the neural population highlight transcriptional regulation, development (particularly Hippo signaling, also in (Piccolo et al., 2022)), and synaptic signaling. Meanwhile, overlapping DEGs with the cortical population highlight cellular proliferation, development, and cellular survival, including WNT signaling that was also highlighted in our previous work looking at transcriptional and epigenetic (K4Me3 ChIP) dysregulation in HD iPSC striatal neurons (Smith-Geater et al., 2020). These data further highlight the contribution of HTT loss of function to HD disease progression.

Interestingly, a subset of the DEGs appear consistent with changes we observed using morphological feature analysis that may help unravel how the gene expression changes relate to cellular changes associated with gain or loss of function that occur because of mHTT. Large sophisticated datasets such as those that are acquired by imaging are perfectly suited to ML applications to find complex signals not readily detectable by conventional approaches (Finkbeiner, 2024) (https://doi.org/10.1016/B978-0-323-95672-7.00009-1). A DNN was previously used to distinguish between CAG-expanded and *HTT* KO from controls in the RUES derivatives that contained densely packed clusters of cells. This finding suggested that there were predictive signals that set apart CAG-expanded and *HTT* KO from control organoids. However, the study did not report the underlying signal nor investigate gene expression changes that could be the basis for the classifier they built (Metzger et al., 2022). By using engineered features, like those used here, the discerning signals that separate groups become significantly more interpretable. Instead of relying on conventional ML approaches, we developed a novel method to track how features change over time, using the MCOT interaction term. This allows us to monitor dynamic cellular-based transformations that could reveal critical disease mechanisms, which might otherwise be overlooked by analyzing images at isolated time points. Similar work has been performed using pseudotime analyses of scRNA data to examine dynamic gene expression changes across time (Hou et al., 2023; Deconinck et al., 2021). But, to our knowledge this has not been done with image data and therefore represents an innovative approach to study disease states.

For future work, one could interweave the MCOT with the OMICs signatures to gain deeper insights into which classes of genes drive cellular processes. Size-related features such as soma and cell area (Fig. 2D, E Supplemental Fig. 6) showed significantly different MCOTs between 72CAG eCNs and controls. The cell area of 72CAG eCNs expanded more rapidly than that of the control eCNs, which remained relatively static. On the other hand, the cell area in *HTT* KO decreased compared to both 72CAG and control eCNs (Fig. 2D, E and Supplemental Fig. 6 A). These morphological changes suggest gain and loss of function mechanisms that align to alterations in gene expression pathways altered such as “negative regulation of cell growth” which is downregulated in the *HTT* KO while upregulated in the 72CAG eCNs (Fig. 3C).

Other morphological changes are consistent with gene expression changes. The majority of “complexity” features all decreased over time all groups, suggesting a decrease in neuronal arborization as the neurons matured. Gene expression changes observed in the class of genes that make up “actin cytoskeletal reorganization”, “negative regulation of cell migration”, and “actin filament organization” were all downregulated in the 72CAG and the *HTT* KO eCNs compared to controls (Fig. 3C). Genes in these pathways drive the underlying forces of organization, structure, and cellular movement and are consistent with the observed morphological changes that may underlie developmental alterations in the 72CAG and *HTT* KO eCNs. Understanding how OMICs alterations caused by the loss of HTT or the presence of the CAG expansion relate to dynamic cellular characteristics could shed light on the mechanisms by which gene expression drives pathological changes in HD and may help decipher gain of function as opposed to loss of function changes. Previous studies have linked small-molecule induced transcriptomic perturbagens to cell morphology alterations (Nassiri and McCall, 2018; Seal et al., 2022). Correlating gene-expression changes to feature-based morphological signatures may be able to help unravel the complexity and timing of when and how cellular systems fail in HD.

A recent study used a similar multi-omic network integration approach to identify links and shared processes between congenital heart disease and autism spectrum disorder (Rosenthal et al., 2023; Rosenthal et al., 2021), which can generate richer hypotheses for assessing cause-and-effect relationships between disease-associated-omics and phenotypes of interest. To begin to assess how nodes in our network influence neurodegeneration, we annotated genes for whether they were essential in iPSC-derived cortical excitatory neurons (Fig. 6E). This analysis identified *RFC2*, *THBS1*, *HAT1*, *PHF6*, *IPO7*, and *COG7* that are each down-regulated in CAG-expanded and *HTT* knockout eCNs and reduce survival upon CRISPRi knockdown (Fig. 6E). This approach is similar to that employed by the NeuroLINCS Consortium (Neuro et al., 2021), where literature mining of ALS-associated genes and functional validation in *Drosophila* showed that nodes in disease-associated networks could be targets for *C9orf72*-mediated neurodegeneration (Neuro et al., 2021). Our analysis provides a platform for nominating pathways and genes that could be targets for therapeutic intervention in HD, and this type of analytical framework can be applied to biological processes involved in multiple neurodegenerative diseases. Future work can use these networks as the basis for implicating genes or proteins in influencing phenotypes such as neuronal survival or the modulation of a proposed biological pathway, nominating them as targets for genetic or pharmacological perturbations. Finally, the feature-based morphological analysis used here may reflect both gain and loss of function properties of the CAG expansions in HTT and could serve as a useful platform for future small molecule, drug or genetic screens that may correct the cellular consequences of mHTT.

## 4. Methods

### 4.1. Pluripotent stem cell culture and sample generation

Isogenic human pluripotent stem cells derived from the RUES2 (NIHhESC-09-0013) embryonic stem cell line were acquired from Coriell through the CHDI foundation.

Lines were maintained on Geltrex basement membrane (Thermo Fisher # A1413301) in mTESR1 medium (Stem Cell Technologies #85850) for omics studies and on Growth Factor Reduced (GFR) Matrigel (Corning # 356231) and mTESR plus medium (StemCell Technologies, # 05825). Cells were grown at 37 °C and 5 % CO2 and were passaged using ReLeSR (Stem Cell Technologies) when they reached 70–80 % confluency. Colonies were cryopreserved in CryoStor CS10 (Stem Cell Technologies). Cell pellets for each of the Omics assays were generated and harvested in parallel for all cell lines, from 3 successive passages using the same lot of each reagent.

### 4.2. Cortical neuron differentiation and sample generation

#### 4.2.1. Omics studies

Pluripotent cells were dissociated to single cells with Accutase (Fisher Scientific # NC9839010) and replated in mTeSR1 plus rock inhibitor (RI) (Peprotech # 1293823) at a density of 4 × 10^6^ cells in laminin (Biolamina #LN521) coated T25 flasks. T25 flasks were fed mTeSR1 for no more than 2 days until they reached >80 % confluence at which point mTeSR was replaced with CM1_o_ consisting of Base C media plus 10μM SB 431542 (Stem Cell Technologies #17502048), 200 nM LDN193189 (Stem Cell Technologies, #72147) and 5μM Cyclopamine (Stem Cell Technologies #72074). Base C media consists of 0.5× DMEM/F12 (Thermo Fisher, #10565042), 0.5× Neuraobasal (Thermo Fisher #21103049), 0.5 % N2 (Thermo Fisher # 17502048) and 1 % B27 (Thermo Fisher #17504–044). From this timepoint (day 0), the cells were fed daily with 10 mL of CM1 until day 8. At d8, cells were passaged 1:2 using cell scraping and gentile trituration onto Poly-ornithine (Sigma # P4957) and 20μg/mL laminin (Sigma Aldrich #L2020) coated T25 flasks. The cells (at this stage, referred to as NPCs) were then fed everyday with CM2_o_ composed Base C media plus 2.5μg/mL Insulin (Thermo Fisher #12585–014), 0.5 % NEAA (Thermo Fisher #11140050), 5 uM Cyclopamine and 20 ng/mL FGF-2 (Peprotech #100–18B). On d15, neural progenitors were dissociated using Accutase and banked in CryoStor CS10. D15 progenitors were banked from 2 rounds of successive passages at the pluripotent stage for each line using identical lots of materials (4–6 batch replicates per line).

Banked progenitors for all lines were thawed in parallel, one batch at a time (4 batches per line), onto Poly-ornithine/laminin 6 well plates at a density of 4 × 10^6^ cells/well in CM3_o_ comprised of Base media C plus 2.5μg/mL Insulin (Thermo Fisher #12585–014), 0.5 % NEAA (Thermo Fisher #11140050) plus 2 uM Cyclopamine, 10 ng/mL BDNF (Peprotech #450–02), and 10 ng/mL GDNF (Peprotech # 450–10). At this point, the cells were considered early-stage cortical neurons (es-CNs) and were fed every day for 6 days. At d21, cells were dissociated using Accutase and replated for final maturation onto Poly-d-lysine (Sigma Aldrich #P6407) coated 12 well plates, at a density of 2–2.5 × 10^6^ cells per well. D21 eCNs were fed every other day for 2 weeks in CM4_o_ comprised of CM3_o_ supplemented with 10μM DAPT, 20 ng/mL BDNF, and 20 ng/mL GDNF. At day35, eCNs were washed 3× with PBS (Thermo Fisher # 10010023), harvested using a cell scraper, and centrifuged to pellet. Transcriptomics and proteomics pellets were flash-frozen dry, epigenetics pellets (ATACseq and ChIPseq) were resuspended in CryoStore CS10 and frozen at −80.

#### 4.2.2. Imaging studies

Pluripotent cells were dissociated to single cells with Accutase (Fisher Scientific # NC9839010) and replated in mTeSR Plus with rock inhibitor (RI) (Selleck Chemicals, #S1049) at a density of 3 × 10^6^ cells per GFR-coated T25 flask. T25 flasks were fed mTeSR Plus for no more than 2 days until they reached >90 % confluence, at which point mTeSR was replaced with CM1i consisting of Base C media plus 10μM SB 431542 (Tocris, #1614), 400μM LDN193189 (Tocris, #605) and 1μM cyclopamine (Tocris #1623). From this timepoint (day 0), the cells were fed daily with 10 mL of CM1 until day 8. At d8, cells were passaged 1:2 using Accutase and plated onto onto Poly-ornithine (Sigma # P4957) and 20μg/mL laminin (Sigma Aldrich #L2020)-coated T25 flasks. The cells (at this stage, referred to as NPCs) were then fed every other day with CM2_i_ until day 15. CM2 consists of: Base C media plus MEM non-essential Amino Acids (Life Technologies, #11140050), 0.1 mM 2-mercaptoethanol, 2.5μg/mL insulin, 1μM cyclopamine and 20 ng/mL FGF. On d15, the NPCs were disassociated using Accutase, and frozen at 8 million cells per vial in Stem Cell Banker cryopreservation solution (CedarLane Labs, # 11890). For each experiment, a vial of each cell line was thawed and plated in CM3 media into a 6-well plate pre-coated with Poly-L-Ornithine and 20μg/uL of laminin at a density of 4 million cells per well. CM3_i_ media consists of Base C media plus MEM non-essential amino acids, 0.1 mM 2-mercaptoethanol, 2.5μg/mL insulin, 1μM cyclopamine, 10 ng/mL BDNF (R&D Systems, #248-BD) and 10 ng/ mL GDNF (R&D Systems, #212-GD. At this point, the cells are considered es-CNs. The es-CNs were fed every day for 6 days until day 21. On day 21, e-CNs were passaged to 384 plates that were precoated with poly-L-ornithine, laminin and fibronectin (CB40008A) in CM4 media. CM4_i_ media consists of 0.5× DMEM/F12, 0.5× Neurobasal, 1× MACS NeuroBrew-21 Supplement (Myltenyi, # 130–093-566), 1× N2 Supplement, 2.5μg/mL insulin rh, zinc solution, 10μM DAPT (R&D Systems, #2634), 20 ng/mL BDNF, 20 ng/mL GDNF and 1× Anti-Anti. After day differentiation day 27, these cells are considered late-stage cortical neurons (eCNs).

### 4.3. Western blot

Cells were broken in a modified RIPA buffer (10 mM Tris-HCl, 150 mM NaCl, 1 mM EDTA, 1 % NP40, 0.5 % SDS, pierce mini protease inhibitor pellet) and allowed to lyse on ice for 10 min. They are then sonicated (3×, 10 s, 40 % amplitude) before being spun down for 10 min at 4 degrees. Protein concentration was quantified using Lowry Protein Assay. 20-25μg protein was used (the amount of protein loaded into the gel was determined previously by the linear range of the antibody). Lysate was run on 3–8 % Tris-Acetate gels (Invitrogen EA03785BOX) with Tris-Acetate running buffer (Fisher Scientific LA0041) and run at 200 V for 45 min at room temperature. Protein was then transferred onto Immobilon-FL PVDF (Millipore Sigma IPFL00010) for 1 h at 10 V. Whole protein was quantified using Revert Total Protein Stain assay (LI-COR Biosciences 926–11,016), and the membrane was blocked with Intercept (TBS) Blocking Buffer (LI-COR biosciences 927–60,010) for 1 h. The membrane was then incubated in anti-Huntingtin primary antibody (Abcam ab109115) overnight, washed three times with TBS-0.1 % Tween-20, and incubated for 1 h in near-infrared-conjugated secondary antibody in intercept block supplemented with 0.1 % Tween-20. Membranes were imaged on a LI-COR scanner.

### 4.4. Immunocytochemistry and microscopy—omics studies

Cells were fixed with 4 % paraformaldehyde (Fisher Scientific # 50980487) for 10 min at room temperature, then washed three times with PBS (Corning # 21030CV). Cells were permeabilized with 0.3 % Triton-X (Sigma #T8787) in PBS for 10 min and then blocked with 2 % goat serum (Thermo Fisher #16210–064), 3 % BSA (Thermo Fisher # 15260–037), 0.1 % Triton-X, and 0.3 M Glycine (Fisher # BP381–1) in PBS for 1 h at room temperature and then incubated in primary antibody diluted in block, overnight at 4 °C (anti-Nestin (1:1000) Millipore MAB5326, anti-FOXG1 (1:300) Abcam ab18259, anti-CTIP2 (1:500) Abcam ab18465, anti-MAP2 (1:1000) Synaptic Systems 188,004, anti-TBR1 (1:250) Abcam ab31940). Primary antibody was removed, and cells washed three times with PBS and then incubated for 1 h in secondary antibody diluted 1:1000 in block, in the dark at room temperature (Alexa Fluor Goat IgG (H+L) Secondary Antibody, Thermo Fisher Scientific). Cells were washed with PBS for three times and then washed in PBS containing Hoechst 33342 (Sigma #14533) for 10 min and then a final wash in PBS. Coverslips were mounted with Fluoromount-G^®^ (Fisher # OB10001) and allowed to dry. Images were acquired on a Keyence BZ-X810 Widefield Microscope. Cell populations were quantified using Cell Profiler (https://cellprofiler.org/) described below (Tian et al., 2019)with n=3-4 images across each differentiation replicate per line.

### 4.5. Immunocytochemistry—imaging studies

eCNs were fixed at day ~35 by permeabilization using a 0.1 % Triton-X/PBS solution for 20 min at room temperature. Permeabilization solution was removed and a 1 M glycine solution added and incubated at room temperature for 20 min. A blocking solution of 0.1 % Triton-X/PBS, 2 % FBS, and 3 % BSA was added after removal of the glycine solution and incubated at room temperature for 1.5 h. Antibodies against: **MAP2** (1:1000 Abcam chicken-anti MAP2 # ab5392), **KI67** (1:200 Millipore mouse-anti KI67 # mab4190), **CTIP2** (1:250, Abcam, #ab 18,645), **TBR1** (1:2000, Abcam Ab183032), **FOXG1** (1:2000, Abcam #ab18259), and DARPP-32 (1:250 abcam, # ab40801) were diluted in blocking solution and incubated overnight at 4 °C. Primary antibody was removed by washing cells 3 times with 0.1 %Triton-X/PBS. Secondary antibodies all from Invitrogen (goat anti chk 647, #A21449; donkey anti-Rb 488, #A32790; goat anti-mouse 488; #A11001 or goat Anti-Rat 488, #A11006) were added to 1:1000 in blocking solution and incubated at room temperature and covered for 1.5 h. Cells were spun down at 8000 RPM in a cold centrifuge during the washes. Cells were washed in PBS for 5 min, then once with PBS plus Hoechst at a dilution of 1:1000 and incubated for 10 min at room temperature, covered. The Hoechst was washed out with PBS and the cells covered in PBS for imaging.

#### 4.5.1. CellProfiler

To measure the patterning propensity for the eCNs, cells were fixed, stained with various antibodies as described above, and images were subjected to a modified CellProfiler (Stirling et al., 2021) pipeline to examine the percent of cells staining for a particular antibody, similar to the colocalization pipeline as described here (https://cellprofiler.org/examples). Images were pooled in CellProfiler and analyzed on a per-image basis. Background values were calculated per image then subtracted from the whole image. Following background correction, all objects in each DAPI image were identified as nuclei using minimum and maximum diameters per object and filtering out excessive intensity values using a minimum cross-entropy thresholding method (Stirling et al., 2021). This method identifies all possible nuclear objects within the appropriate size range. Intensity values were calculated per object then used to filter out non-nuclear objects or dead cells that display bright nuclei, and then relabelled as nuclei segments. Images that contained KI67/ TBR1/ FOXG1 /CTIP2 or DARPP32 staining were subjected to a feature enhancement step that increases the signal-to-noise ratio. Enhanced images were run through segmentation to identify all objects considered “positive” in terms of size and signal intensity again using a minimum cross-entropy thresholding method (Stirling et al., 2021). These identified objects were renamed as “positively labeled” segments. These segments were related to the nuclei segments to determine how many nuclei were “positive” for that particular antibody. Finally, the number of “positively labeled” cells were divided by the number of total nuclei to calculate the percent of KI67/ TBR1/ FOXG1/CTIP2 or DARPP32 “positive” cells. The segmentation overlays and math were exported from the program and then plotted in Prism10 (Dotmatics, 2023).

Cell Profiler was also used to measure neurite length was based on Tian et al. 2019 (Tian et al., 2019), with some additional modifications to the workflow as previously described (HD iPSC Consortium, 2017). The same background correction and primary object identification as the analysis described above was performed but on images from differentiation day ~32 from live cells that express SYN:EGFP. The soma for each neuron was identified in CellProfiler (Stirling et al., 2021) and segmented based on size, eccentricity, and intensity of SYN:EGFP signal. Soma segments were filtered by eccentricity to eliminate any potentially dead, rounded cells or debris from the analysis. Next, the images were run through feature enhancement to increase contrast between potential neurites and the background. Neurites were identified using the filtered soma masks as points of origin for neurite outgrowth and an Otsu thresholding method (Stirling et al., 2021). Skeleton length as well as trunk and branch end numbers were calculated and exported as a spreadsheet. We normalized each experiment to account for variation across batches of differentiated cells, as previously described (HD iPSC Consortium, 2017). Briefly, we calculated the average neurite length of control eCNs for each batch and normalized each individual value by this average to generate an averaged ratio for each neurite measured. These ratios are used in the histogram in Fig. 2.

### 4.6. Statistical analysis

We developed a statistical package called RMeDPower (Shin et al., 2022) in R, a complete package of statistical tools that allow a scientist to understand the effect size and variance contribution of a set of variables within a dataset to a given response (Shin et al., 2022). RMeDPower uses linear mixed models on repeated measures data such as those described here. Outliers were removed using the Cooks distance (Cook, 1979) and data was log transformed for statistical analysis. All p values and estimates for each comparison for the neurite analysis and percent positive staining were calculated in RMeDPower (Shin et al., 2022).

### 4.7. RNAseq

RNA was processed as previously described (Neuro et al., 2021) from the 15 pluripotent samples (5 lines in triplicate) and 16 cortical neuron samples (3 replicates for all lines except *HTT*KO which had 4 replicates as the 3rd replicate for proteomics and epigenomics assays were derived from separated differentiation batches due to insufficient yield). Briefly, the RNeasy mini kit was used along with QIAshredders for homogenization and DNase I for gDNA elimination (Qiagen #74106, # 79656, #79254). The RNA samples were then analyzed for an RNA integrity number (RIN) which was determined to be greater than 9 for each sample except for one, which had RIN of 7.3 (which later passed QC at the library stage). rRNAs were removed and libraries generated using TruSeq Stranded Total RNA library prep kit with Ribo-Zero (Qiagen). RNA-seq libraries were titrated by qPCR (Kapa), normalized according to size (Agilent Bioanalyzer 2100 High Sensitivity chip) and sequenced by NovaSeq6000 using the S4 flowcell with sufficient paired end (PE) sequencing cycles to obtain 50 million PE reads per sample.

#### 4.7.1. RNAseq data analysis

Sequenced reads were trimmed for adaptor sequence or low-quality sequence using Trimmomatic (Bolger et al., 2014) and aligned using HISAT2 using an index created from Ensembl GRCh38 release 104. Gene counts were quantified using featureCounts (Anders et al., 2015) and differential analysis was performed DESeq2 (Love et al., 2014). Additional QC metrics were obtained using SAMtools. Gene Ontology analysis of the top 50 most variable genes contributing to the cortical stage PCA was analyzed using g:Profiler (Kolberg et al., 2023) using the entire human genome as the background.

### 4.8. Epigenetic data

#### 4.8.1. ATAC-seq

We used 50,000 nuclei for the transposase reaction, following the method described previously (Corces et al., 2017). Subsequently, samples were purified with the DNA Clean & Concentrator–5 Kit (Zymo Research), and then PCR-amplified using Nextera indexing primers (Illumina). The number of PCR cycles were optimized by qPCR to avoid over amplification of the libraries. The final libraries were purified using 1× PCRClean DX beads (Aline Biosciences). The enrichment of accessible chromatin regions in the libraries were assessed by qPCR using primers mapped to open chromatin region and gene desert regions (Milani et al., 2016). The libraries fragment distribution was analyzed by a Fragment Analyzer^™^ instrument (Advanced Analytical), and the concentration measured by a qPCR-based method (KAPA Library Quantification Kit for Illumina Sequencing Platforms). PE sequencing (40 nt) was conducted using Illumina NextSeq platform at the MIT BioMicroCenter. The quality of the data was assessed using FastQC and the sequences aligned to the HG38 reference genome build using BWA, a short sequence aligner, with default parameters. Aligned reads with a mapping quality of less than 10 and mitochondrial reads were removed using SAMtools. Duplicate reads were removed using PicardTools.

#### 4.8.2. ChIP-seq

Histone modification ChIP experiments were performed using antibodies against H3K27me3 (C36B11, Cell Signaling), H3K4me1 (Abcam # ab8895,), H3K4me3 (Millipore #07–473), H3K27ac (Cell Signaling # D5E4) and IgG. For each ChIP, 1 × 10^6^ cells were incubated in lysis buffer (50 mM Tris-HCL pH 8.0, 150 mM NaCl, 1 % Triton X-100, 0.1 % Na- deoxycholate, and 5 mM CaCl2 supplemented with protease inhibitors and 10 mM sodium butyrate for K27ac ChIP) for 20 min on ice. The chromatin was digested to 1–5 nucleosomes using Micrococcal Nuclease (NEB # M0247S), at concentration determined by titration experiments, for 10 min at 37 °C. To terminate the digestion, 20 mM EDTA was added to the samples. The antibody was added and incubated overnight at 4 °C. On the next day, 25μl of protein G Dynabeads (Thermo Scientific # 10004D,) was added and the samples rotated for 2 h at 4 °C. The beads were washed 6 times: 2 washes with RIPA buffer (10 mM Tris-HCl pH 8, 1 mM EDTA, 140 mM NaCl, 1 % Triton X-100, 0.1 % Na-deoxycholate, 0.1 % SDS), 2 washes with high salt buffer (10 mM Tris-HCl pH 8, 1 mM EDTA, 360 mM NaCl, 1 % Triton X- 100, 0.1 % Na-deoxycholate, 0.1 % SDS), 2× washes with LiCl buffer (10 mM Tris-HCl pH 8, 1 mM EDTA, 250 mM LiCl, 0.5 % IGEPAL CA-630, 0.5 % Na-deoxycholate) and 1 wash with TE (10 mM Tris-HCl pH 8, 1 mM EDTA). The DNA was eluted by incubation of the beads in elution buffer (10 mM Tris-HCl pH 8, 5 mM EDTA, 300 mM NaCl, 0.1 % SDS, 50μg Proteinase K) for 1 h. at 62 and purified with 1× PCRClean DX beads (Aline Biosciences). The sequencing libraries were constructed using the NEBNext^®^Ultra^™^ II DNA Library Prep Kit for Illumina (NEB # E7645S), followed by analysis using Fragment Analyzer^™^ instrument (Advanced Analytical), and quantification by a qPCR-based method (KAPA Library Quantification Kit for Illumina Sequencing Platforms). Single-end sequencing (50 nt) was conducted using Illumina HiSeq 2000 or Nova-Seq S4 platform at the MIT BioMicroCenter. QC of the sequences, genome alignment and removal of low-quality reads and duplicates were done as described for ATAC-seq. Regions marked by the specific histone modification (peaks) with MACS2 were determined using the “broad peak” setting, a “broad” cutoff of 0.1, a q- value cutoff of 0.05, and the aligned reads from an IgG ChIP sample as a control.

#### 4.8.3. Processing epigenetic data

We processed the ATAC-seq data using the ENCODE-DCC ATAC-seq pipeline v.1.7.1. Using this pipeline, we identified FASTQ read adaptors with the detect_adapter.py script in GGR_code (https://github.com/nboley/GGR_code). We trimmed read adaptors with the trim_a-daptors function in cutadapt 1.9.1. Processed FASTQs, were aligned using Bowtie2 version 2.2.6, requiring SAMtools version 1.7 and SAM-stats version 0.2.1. Picard tools version 1.126 MarkDuplicates was used to deduplicate the aligned reads. These tools were used with default parameters except for setting the parameters “atac.auto_adect_adapter” and “atac.enable_xcor” to “true”.

The ChIP-seq data was analyzed using the ENCODE-DCC ChIP-seq pipeline v1.3.6. In this pipeline, FASTQ data were cropped with Trimmomatic 0.39, aligned with Bowtie2 version 2.26 and deduplicated with Picard version 1.126 MarkDuplicates. Default parameters were used for all arguments except for setting “chip.pipeline_type” to “histone”.

#### 4.8.4. Analysis of epigenetic data

From the aligned, deduplicated ATAC-seq data, MACS2 version 2.1.0 was used to identify peaks that had significant local enrichments at a p-value threshold of 0.01. For the aligned, deduplicated ChIP-seq data, peak calling was performed using SPP version 1.14, GEM 2.4.1 and PeakSeq version 1.25. IDR 2.0.4 was run to determine a high-quality set of consensus peaks from the results of the multiple peak callers. Upon identifying peaks, DiffBind version 2.10.0 was used to perform differential analysis between ChIP-seq or ATAC-seq peaks for the following comparisons: *HTT* KO vs control, 56CAG vs control, and 72CAG vs control. We refer to the union of peaks found in 20CAGn1 and 20CAGn2 cells as “control” in these comparisons. DiffBind in R version 3.5.2 was used to identify differential peaks as those with an FDR-adjusted p-value less than 0.1.

To determine similarities and differences across cortical neuron replicates and genotypes in the ATAC-seq or ChIP-seq data, Principal Component Analysis (PCA) was used as implemented in Scikit-Learn version 0.23.2. To preprocess the data, peaks were limited to those that were within 5 kb from a protein-coding gene. The signal of the filtered peaks was standardized before performing PCA with 10 principal components. Replicates were removed from the analysis if they failed the ATAC-seq or ChIP-seq ENCODE benchmarks determined from FastQC (http://www.bioinformatics.babraham.ac.uk/projects/fastqc/).

We determined enriched, known motifs from the ATAC-seq and ChIP-seq marks by applying HOMER version 4.11 to the significantly differential ATAC-seq or ChIP-seq peaks inferred from DiffBind (http://bioconductor.org/packages/release/bioc/html/DiffBind.html). The background for the motif enrichment analysis was the set of all peaks identified by MACS2 for a given genotype, across replicates. For motif enrichment, we looked for motifs in 200 base-pair regions in repeat-masked sequences.

### 4.9. Proteomics

Upon receipt, 21 cortical neuron samples from 7 lines (3 replicates of each) were lyophilized and lysed in Tris-Urea lysis buffer (33 mM Tris, 1.33 mM CaCl2, 4 M urea, 0.67 mM DTT in 1 M NH4CO3, pH 8.3–8.7) with sonication. Protein BCA assay was performed, and a volume required for sufficient protein was taken from the cell lysate for the proteomic sample preparation on Beckman Biomek i7 Automated Workstation. The samples were reduced with 50 mM TCEP, alkylated with 200 mM iodoacetamide (IAA), digested by Trypsin/LysC, followed by the peptide desalting by using Waters Oasis HLB plate. Desalted tryptic peptides were dried down in a SpeedVac and reconstituted with 0.1 % aqueous formic acid for data independent acquisition (DIA)-mass spectrometry (MS) analysis.

#### 4.9.1. LC-MS analysis

The peptide samples were separated on a 45-min gradient, a window of 15 *m/z*,on the UltiMate 3000 HPLC system (Thermo Scientific) connected with Orbitrap Fusion Lumos Tribrid mass spectrometer (Thermo Scientific). Here, mobile phase A and B consisted of 0.1 % formic acid in water and 0.1 % formic acid in 100 % acetonitrile, respectively, using a gradient of 6–36 % B in 41 min, 36–80 % B in 1 min, and 80 % B in 1 min at the flow rate of 9.5 uL/min. Resolved peptides were ionized by an EASY-Spray ion source. In MS scans, internal mass calibration was performed using EASY-IC. Mass spectra were acquired in a data-independent manner, parameters for the DIA method are as follows: resolution at 60,000, mass range of 400–1000 m/z, and maximum injection time of 50 ms for MS1 scan; resolution at 15,000, HCD collision energy of 30 %, mass range of 400–1000 m/z, and maximum injection time of 30 ms for MS2 scan. For both MS1 and MS2, RF Lens 30 % and normalized AGC Target 150 % were applied.

#### 4.9.2. Proteomics data analysis

MS data files were analyzed in DIA-NN v.1.8 (Demichev et al., 2020), using the spectral library-free search feature with Prosit against the Human Uniprot database. For cohort comparisons of cortical neuron samples, the DIA-NN generated protein intensities were log base 2-transformed and standardized across each sample so that the protein quantity distribution in each sample had a mean of zero and standard deviation of one. Proteins quantified in every sample were compared between the control groups and expanded CAG or KO groups respectively using t-tests with Benjamini-Hochberg correction.

### 4.10. Longitudinal single cell imaging and analysis

eCNs were transduced with pHR-hSyn:EGFP (Addgene #114215) (Keaveney et al., 2018) or GEDI (Linsley et al., 2021) at ~5 MOI after plating in 384-well format. eCNs were subjected to RM and imaged daily as previously described (HD iPSC Consortium, 2012; Arrasate and Finkbeiner, 2005; Arrasate and Finkbeiner, 2012; Arrasate et al., 2004; Barmada et al., 2010; Miller et al., 2011; Mitra et al., 2009b; Tsvetkov et al., 2013; Baxi et al., 2022; Germain and Testa, 2017; Mitra et al., 2009a; Shaby et al., 2016) using ImageXpress Micro Confocal High-Content Imaging System from Molecular Devices for 7–10 days starting at differentiation day ~28–32. Images of different microscope fields from the same well were stitched together into montages, and montages of the same well collected at different time points were organized into composite files in temporal order. Image analysis was performed in a computational pipeline constructed within the open-source program Galaxy, to identify individual cells to perform survival analysis, or apply morphological measurements as described below. eCNs were hand tracked using previously described methods and a Cox mixed effects proportional hazards analysis (Therneau, 2020). We compared the survival to the group of 20CAG lines (20CAGn3 and 20CAG44) to the 72CAG lines (72CAGn2, 72CAGn3 and 72CAGn1). We also compared the survival rates of the control compared to the KO eCNs.

#### 4.10.1. Feature extraction

After eCNs were subjected to RM as described above, images from control 20CAG, expanded 72CAG and KO lines were processed in our custom-built image-processing pipeline Galaxy software (Afgan et al., 2018). Images from the same well were stitched together into montages, and cells were identified and segmented using a thresholding method based on the standard deviation from the mean to ensure that brighter cells are accurately identified and isolated. Images were subjected to our custom-built filtering algorithm written in Python to crop each detected object into 300 × 300 pixel size and remove any crop with edge artifacts, dead cells, clumps, out-of-focus, images with bad intensity signal, or non-centered cells to ensure cell quality for further analysis. These are called “cell crops”. This was achieved through a series of well-defined steps. First, cells located on the image edge that could not be cropped into 300 × 300 pixel region were excluded. The remaining cells were cropped and subjected to a Gaussian blur algorithm (Gedraite and Hadad, 2011) to smooth the image and reduce high-frequency noise to feed into Otsu thresholding (Otsu, 1979), which separates objects from background. These segmented objects were then subjected to the Erosion and Dilation algorithm (Haralick et al., 1987), which acts to shrink and enlarge image foreground to detect and separate adjacent cells and eliminate small artifacts. Images with more than one potential cell were filtered out to ensure our analysis only focused on individual cell images for single cell analysis. In addition, cells that fell outside the defined range of minimum and maximum area were also filtered out. This helps to exclude cells that were either too small to be of interest, often indicating artifacts or dead cells, or too large, suggesting the presence of clumps. The cell crops were then fed into our custom-made feature extraction algorithm. The feature extraction algorithm is written in Python and contained within a Jupyter notebook (https://github.com/finkbeiner-lab/FeatureAnalysis)-.

Morphological properties such as cell area, cell texture, complexity and intensity were analyzed by the following steps. Various features such as median value, and distribution skewness statistics were extracted using a combination of image processing packages, notably OpenCV (GitHub, 2023), NumPy (Harris et al., 2020), Scikit-Image (van der Walt et al., 2014), and SciPy (Virtanen et al., 2020) library. To obtain these features, a median blur filter (Davies, 2004) preprocessing was applied to the image crops. Subsequently, a binary threshold process was employed to selectively capture only the soma (the cell body of each neuron) for analysis. This generates binary masks which were then used to calculate key statistics of each soma determined by the total pixel count within the mask; the Soma Median, representing the median pixel value of the soma area; and the Soma Skewness, which measures the asymmetric in the distribution value within the soma area. Additionally, cell area was extracted, which is the total pixel count of both the soma and neurite areas, to capture valuable information about the entire cell structure. A mean blur filter (Davies, 2004) feature was used to smooth the image to reduce unnecessary noise. The median of the local gray-level histogram from the mean filter was calculated to provide insights into the image intensity of each cell line.

To analyze the shape and the morphology of the cells, edge detection methods were used to try to identify any distinct edge characteristics, such as Sobel operator (Uppala and Sahr, 1997), HOG (histogram of gradients) (Li et al., 2016), Canny edge detection (Canny, 1986) and Fast Fourier Transform (FFT) (Kaso, 2018). The Sobel operator (Uppala and Sahr, 1997) was used to capture the mean, the median value, and the noise in the image, which highlight the areas of significant intensity changes in the cell image and are indicative of the presence of edges. HOG (Li et al., 2016), on the other hand, captures more information within the cells and more distinctive local patterns. Canny edge detection was used to capture sharp and continuous edges in the cell, and a High-Pass Filter (HPF) using Fast Fourier Transform (FFT) (Kaso, 2018) were applied to enhance high-frequency components, including edges and texture patterns.

To quantify the complexity in the cell, Minkowski-Bouligand Fractal Dimension (Squarcina et al., 2015) feature was used to measure the roughness or complexity of an object. There are also two texture features including Haralick (Haralick et al., 1973) and GLCM(Gray-Level Co-occurrence Matrix) (Iqbal et al., 2021) features that quantify the spatial relationships between pixel intensities in an image. The GLCM Correlation measures the probability occurrence of neighboring pixels in the horizontal and vertical directions, the GLCM Energy is the sum of the squared elements and captures the uniformity. The GCLM Correlation measures the probability of the neighboring pixel pairs (Mutlag et al., 2020; Park and Guldmann, 2020).

The GLCM measures subtle texture differences between cell lines that may not be apparent through other features. To analyze the branching patterns, features such as Frangi Filter (Frangi et al., 1998), a vessel enhancement filter, and Sholl Intersections (Kutzing et al., 2010) were used. Both can provide valuable information about neurites and the pattern of neurite outgrowth.

Values from all the features were extracted from the first (T1) and last timepoint (~T7) available were saved into a CSV file for further downstream analysis. The multiple-timepoint data was combined and processed through a RMedPower (Laird and Ware, 1982) tool kit that allows a user to examine the normality assumptions for the linear mixed model (Laird and Ware, 1982) and adjust if necessary as well as using the Rosner’s test (Rosner, 1983) to remove any detected outliers on a per-feature basis. All of the feature values except for “edge” and “sholl med” were not normally distributed so they were log transformed as described (Laird and Ware, 1982). To estimate this shift modulated by disease status we use a Linear Mixed Effects Model (LMM) (Kuznetsova et al., 2017; R Core Team, 2012) called RMedPower (Laird and Ware, 1982) to capture the mean feature values in control 20CAG, expanded 72CAG and *HTT*KO lines at each distinct timepoint and the additional shift across the two time-points due to a change in disease status via an interaction term. Hence, the interaction term is between the time and the disease status variables. The mathematical formulation of this LMM is described next.

Let $Y_{ijk}^{t}$ denote the feature value in the natural or the logarithm scale (see above) of the $k^{\text{th}}\left(k\;\epsilon \left\{1,2,\cdots ,N_{ij}^{c}\right\}\right)$ cell at time-point $t(t\;\epsilon \left\{T|1|,|T|7\right\})$, derived from the $j^{\text{th}}\left(j\;\epsilon \left\{1,2,\cdots ,N^{c}\right\}\right)$ cell-line assayed in the $i^{\text{th}}\left(i\;\epsilon \;Exp=\left\{1,2,\cdots ,N^{e}\right\}\right)$ experimental batch. $N^{e}$ is the number of experimental batches. $N^{c}(5)$ is the number of cell-lines – 2 20CAG lines, 3 72CAG lines and 1 HTTKO line. $N_{ij}^{c}$ is the number of cells from cell-line j assayed in experimental batch i. The notation A and R refers to the sets of all alternate and reference cell-lines respectively that are used in the experiment. Then the LMM states,

$$Y_{ijk}^{t}=Z_{i}+W_{j}+\beta_{0}+\beta_{1}I_{alt}(j)+\beta_{ref}I_{T7}(t)+\Delta \beta_{alt}I_{T7,alt}(t,j)+\varepsilon_{ijk}$$

$$Z_{i}\sim N\left(0,\sigma_{i}^{2}\right)$$

$$W_{j}\sim N\left(0,\sigma_{j}^{2}\right)$$

$$\varepsilon_{ijk}\sim N\left(0,\sigma^{2}\right)$$

$$I_{T7}(t)=1\;\text{if}\;t=T7$$

$$I_{T7}\left(t\right)=0\;\text{if}\;t=T1$$

$$I_{alt}(j)=1\;\text{if}\;j\;\epsilon \;\text{A}$$

$$I_{alt}(j)=0\;\text{if}\;j\;\epsilon \;\text{R}$$

$$I_{T7,alt}(t,j)=1\;\text{if}\;t=T7\;\text{and}\;j\;\epsilon \;\text{A}$$

$$I_{T7,alt}(t,j)=0\;\text{if}\;t=T1\;\text{or}\;j\;\epsilon \;\text{R}$$

Where $Z_{i}$ denotes the random effect associated with the $i^{\text{th}}$ experimental batch, $W_{j}$ denotes the random effect associated with the $j^{\text{th}}$ cell-line. Both these random effects are assumed to drawn from normal distributions with zero means and variances $\sigma_{i}^{2}$ and $\sigma_{j}^{2}$ capturing the inter-experimental batch variances and inter-cell-line variances, respectively. The $\varepsilon_{ijk}$ term captures the residual error of the model. Let $\beta_{\text{ref}}$ refer to the change in the marginal expectation of the feature value over time for the reference cell-lines (e.g., 20CAG), i.e.,

$$\beta_{\text{ref}}=E_{i\;\epsilon \;Exp,j\;\epsilon \;R,k\;\epsilon \;N_{ij}^{c}}\left[Y_{ijk}^{T7}\right]-E_{i\;\epsilon \;Exp,j\;\epsilon \;R,k\;\epsilon \;N_{ij}^{c}}\left[Y_{ijk}^{T1}\right]$$

The $\beta_{0}$ term captures the mean feature value at time T1 for the reference cell-lines, i.e.,

$$\beta_{0}=E_{i\;\epsilon \;\text{Exp},j\;\epsilon \;R,k\;\epsilon \;N_{ij}^{c}}\left[Y_{ijk}^{T1}\right]$$

The $\beta_{1}$ term captures the change in mean feature value at time T1 between the alternate and the reference cell-lines, i.e.,

$$\beta_{1}=E_{i\;\epsilon \;Exp,j\;\epsilon \;A,k\;\epsilon \;N_{ij}^{c}}\left[Y_{ijk}^{T1}\right]-E_{i\;\epsilon \;Exp,j\;\epsilon \;R,k\;\epsilon \;N_{ij}^{c}}\left[Y_{ijk}^{T1}\right]$$

The subscripts for the expectation function, E indicates that the mean is taken by averaging out effects over all experimental batches, over all reference cell-lines and all cells in each of these reference cell-lines. $I_{T7}(t)$ and $I_{T7,\text{alt}}(t,j)$ are indicator functions. $\beta_{\text{alt}}$ can analogously defined for the alternate cell-line (e.g., 72CAG) though is not directly estimated as one of the coefficients in the above model. $\Delta \beta_{\text{alt}}$ estimated by the coefficient of the interaction term in the LMM described above, captures the additional change over time of the mean feature value for the alternate cell-line. We call this interaction term the “Morphology Change Over Time” or “MCOT”(the time:disease_status interaction terms when scaled by the change over time in the 72CAG and *HTT*KO or control and expressed as percentages (Fig. 2E). Specifically, MCOT is defined as,

$$\text{MCOT}=100\times \frac{\Delta \beta_{\text{alt}}}{\beta_{\text{ref}}}=100\times \frac{\left(\beta_{\text{alt}}-\beta_{\text{ref}}\right)}{\beta_{\text{ref}}}$$

Significant differences in the rate of change of these features were identified by the significance of the interaction term. The LMMs were fitted using the lmerTest (Kuznetsova et al., 2017; R Core Team, 2012).

Use of RM allows us to acquire very large sample sizes and therefore resulted in many observations, we observed very low p-values when comparing most all of the features, but in some cases this change was very small therefore, we considered the effect sizes in highlighting significant associations (Bilican et al., 2012) and only considered a change of 15 % or greater to be considered a bona fide difference across the groups.

#### 4.10.2. Identifying concordant changes in differential omics data

To define differentially expressed genes in the RNA-seq data, we used an FDR-adjusted p-value cutoff of 0.05 and an absolute log_2_ fold change cut off of 1. For the epigenetic data, we used an FDR-adjusted p-value cutoff of 0.1. For the proteomics, we used a nominal p-value cut off of 0.05 and an absolute log_2_ fold change cutoff of 0.6. The different log_2_ fold change cutoffs for proteomics and transcriptomics was inferred empirically from a previous study that used a similar approach for measuring transcriptomic and proteomic data from stem cell-derived models (Neuro et al., 2021). To identify enriched pathways, we ranked each omics data by their log_2_ fold change and applied Gene Set Enrichment Analysis (GSEA) for each assay. As a reference pathway set, we used the 2021 Gene Ontology Biological Processes, as obtained from Enrichr at https://maayanlab.cloud/Enrichr/#libraries. To apply GSEA to proteomic data, we limited the background set of genes to proteins that were detected in the proteomics analysis to account for the observation that not every gene in the genome is detectable by mass spectrometry.

For each assay, we defined “concordant” changes as a differentially altered gene or protein in at least one of the HD or KO comparisons that had the same fold change direction in all three genotypes. For example, if gene A were differentially expressed in the RNA-seq data when comparing *HTT*KO to control, we would define the change as concordant if gene A was downregulated when comparing *HTT*KO to control, 56CAG to control and 72CAGn1 to control (20CAGn1 and 20CAGn2). This analysis was performed separately for each assay. To assess whether the set of differentially expressed genes or differentially abundant proteins were enriched for genes or proteins with concordant changes, we performed a hypergeometric test, where the background was either the total number of genes that were expressed in the cortical neuron data or the total number of proteins detected. Visualizations were performed in R using the packages ComplexHeatmap version 2.4.3 and UpSetR version 1.4.0. For GSEA, we used the R package fgsea version 1.14.0.

### 4.11. Multi-omic integration analysis for HTT KO and HD cell lines

To identify the shared and distinct biological processes associated with *HTT* loss-of-function and HD. We utilized the Prize-Collecting Steiner Forest algorithm (PCSF) as implemented in OmicsIntegrator 2 (v2.3.10, https://github.com/fraenkel-lab/OmicsIntegrator2^92^). The PCSF algorithm identifies genotype-associated networks based on retrieving significantly altered omics signals without including low-confidence edges. The result includes the differentially altered omics and predicted interactors from the reference interactome, which we call “predicted nodes”. We used OmicsIntegrator to map proteomic, transcriptomic, and epigenetic motifs to a set of known protein-protein and protein-metabolite interactions derived from physical protein-protein interactions from iRefIndex versions 17 and 14 as well as and protein-metabolite interactions described in the HMDB and Recon 2 databases.

We performed differential analyses comparing the *HTT* KO line to the combined controls, 56CAG to the controls, and 72CAG to the controls. We applied different significance thresholds for each data type so our output network would not be overrepresented by a single data type. Differentially expressed genes were determined as those with an FDR-adjusted p-value less than 0.1 and absolute log_2_ fold change greater than 1 after applying DESeq2 to the transcriptomics data (Love et al., 2014). Differential proteomics were those with an FDR-adjusted p-value less than 0.1 determined by a t-test after normalization. We relaxed the log fold change threshold for network integration to better infer biological processes that involve proteomic changes in the context of CAG repeat expansion or *HTT* knockout. Enriched motifs were determined as those with an FDR-adjusted p-value after motif enrichment analysis using HOMER (Heinz et al., 2010). To set the weights, or prizes, of the termini, we associated each differentially expressed gene, differentially abundant protein or enriched epigenetic motif with its negative log_10_, FDR-adjusted p-value. Prize values were minimum-maximum scaled by data type. For interpretability, we kept a proportion of the most enriched prizes for network integration; for the *HTT* KO network, the top 30 transcriptomic, proteomic and epigenomic prizes were included, and for the expanded CAG network the top 50 transcriptomic, proteomic and epigenomic prizes were selected from the 56CAG and 72CAG prizes. For the expanded CAG network, to reflect a greater degree of confidence in the proteomic and transcriptomic data than the epigenetic data, we weighted the epigenetic prizes by a factor of 0.33. The same methods for multi-omic integration were used for the ES and the cortical neuron networks.

To assess the robustness and specificity of our networks, we randomized the edges and prizes of our network solution, respectively, adding gaussian noise to the randomized edges. Nodes that were not previously assigned a prize (termed Steiner nodes) were filtered out if they appeared in fewer than 40 edge randomizations or more than 40 prize randomizations. Louvain community detection was then performed to create sub-network clusters. Within each sub-network, gProfiler2 version 0.2.0 (Kolberg et al., 2020) was used to identify significantly enriched biological processes via Gene Ontology Biological Process terms or REACTOME pathways. To identify the overlap between the KO and expanded CAG networks, we took the union of nodes and edges between the two networks and re-clustered the networks based on those nodes that were only found in the KO network, nodes found only in the expanded CAG network and the nodes found in both networks.

OmicsIntegrator requires selecting hyperparameters to visualize a genotype-specific network. These hyperparameters are the weights on the prizes (b), the network size (w) and the edge penalty (g). To choose a hyperparameter set, OmicsIntegrator was run on a set of parameters: β={2,5,10},ω={1,3,6} and γ={2,5,6}. The parameter choices were evaluated based on the mean node robustness and specificity, minimizing the KS statistic between prize and Steiner node degree, and the networks with high ratios of proteomic and transcriptomic prizes to epigenomic prizes. Networks were visualized using Cytoscape version 3.8.0.

### 4.12. Resource availability

Materials and protocols will be distributed to researchers in a timely manner following publication. All data has been deposited in public data bases: RNAseq, ATACseq and ChIPseq in GEO, Protein in Massive. ATACseq data and ChIP-seq data sets for the ES cells have been deposited in GEO with accession number GSE282628 and the epigenetic data for the cortical neurons in GEO with the accession number GSE282626. RNAseq data for the ES cells have been deposited in GEO with the accession number GSE284565 and the accession number GSE284798 for the cortical neurons. Proteomics has been deposited in Massive with accession number MSV000096708 for the ES cells and MSV000096707 for the cortical neurons.

This study did not generate new unique reagents. Further information and requests for resources and reagents should be directed to and will be fulfilled by the lead contact.

## Supplementary Material

1

2

3

4

5

6

7

## Data Availability

The data is available through public repositories

## Appendix A. Supplementary data

Supplementary data to this article can be found online at https://doi.org/10.1016/j.nbd.2025.106914.
