# Supplementary material for: Intersecting impact of CAG repeat and huntingtin knockout in stem cell-derived cortical neurons
Source: Neurobiol Dis. Author manuscript; Available in PMC 2025 Dec 1. (PMC12668206; doi:10.1016/j.nbd.2025.106914)

**Supplemental Figure 1: Western blot analysis of HTT expression.**

Protein pellets were collected at the ES stage and after eCN generation to confirm HTT knockdown in the *HTT* KO line and to distinguish the two different alleles (control length and highly expanded) for the 72CAG line on a western blot probed with an N-terminal targeting HTT antibody (Abcam ab109115). **A)** ES stage *HTT* KO, 20CAGn2, and 72CAGn1 lines probed for HTT (top) and total protein stain (bottom). Ladders shown are the LI-COR Chameleon Duo Ladder (left) and Tri-color Prestained Protein Marker II (Bioland Scientific). **B)** eCN differentiated *HTT* KO, 20CAGn2, and 72CAGn1 lines probed for HTT (top) and total protein stain (bottom). Ladder shown is the LI-COR Chameleon Duo Ladder (left).

**Supplemental Figure 2:** **eCNs subjected to RM express cortical forebrain markers.** At differentiation day ~35 eCNs were fixed and stained with antibodies directed at various cell type specific markers from all clones (*HTT* KO, 20CAGn3, 20CAGn4, 72CAGn2, 72CAGn3, 72CAGn4). Representative immunofluorescence images showing expression of the neuronal forebrain markers **A)** TBR1 **B)** BCL11B **C)** FOXG1. **D)** eCNs also patterned into cells that express the inhibitory GABAergic marker DARPP32. **E)** eCN cultures contained some proliferating cells as shown by the Ki67 stain. All cells stained with Hoescht (blue) nuclear marker. Panels **A-D** were all green with MAP2 (red). Scale bar = 220mm.

**Supplemental Figure 3: Quantification of day ~35 eCNs reveals a substantial proportion of neurons expressing typical forebrain markers.**

Day ~35 eCNs were fixed and stained with antibodies and Hoescht and imaged using RM. Images were subjected to a modified Cell Profiler pipeline as detailed in a previous study(Wang et al., 2024) to quantify the proportion of cell expressing each marker. We used a Linear Mixed Effects Model (LMM)(Kuznetsova et al., 2017; R Core Team, 2012) to quantify the percentage of cells positive for each antibody across each group. **A**, **C**, **E**, **G**, **I)** histograms showing the percentage of positive cells per image tile for each Hoechst- stained nuclei. **B**, **D**, **F,** **H, J)** histograms showing each group combined after outliers were removed as determined by the Cooks distance(Cook, 1979). Each dot represents the percent positive fraction of antibody- labeled cells measured from an entire image.

**Supplemental Figure 4: Image and feature analysis pipeline. A)** Example micrograph of live eCNs transduced with synapisn: EGFP imaged by RM every 24 hours. Scale bar = 65 mm. **B)** Cumulative risk of death curve plotted to show the variation across experiments. Each experiment is plotted by a different line thickness as delineated in the panels to the right. The average across all experiments are shown in bold.  **C)** Example of image processing pipeline to generate single cell crops for ML. Each original image is acquired using RM. Images are subjected to our custom- built Galaxy pipeline that performs a background subtraction step, followed by segmentation based on the pixel intensity of each fluorophore. Next small or large objects are filtered to obtain single cells. These cells are then cropped to a 3 x3 image which is used for feature analysis. **D)** Example of feature maps shown for a single cropped neuron.

**Supplemental Figure 5: Comparison of eCN features at a single timepoint. A)** The perimeter of neuron including processes within each crop as captured by the Edge feature was larger in the 72CAG as compared to 20CAG and *HTT* KO. **B)** The shape or roundness of each cell as captured by Eccentricity showed that the 72CAG eCNs were rounder than the controls. **C)** The median number of processes emanating per soma captured by the Median Sholl feature was significantly different in the *HTT* KO eCNs as compared to control and 72CAG eCNs. Measurements were taken on images from differentiation day 28.

**Supplemental Figure 6: Summary plots displaying the residual feature changes over time. A)** The cell area feature captures the total cell area within each cropped image. The cell area changed a greater amount in the 72CAG eCNs compared to controls, whereas the cell area decreased in the HTT KO eCNs over time. **B)** The soma area feature captures the size of just the soma. All somas grew smaller over time, but at different constants. **C)** The edge feature captures the perimeter of each neuron including processes within each crop. The MCOT for edge was larger in the control compared to 72CAG and *HTT* KO eCNs. **D)** The eccentricity feature captures the shape or roundness of each cell. The eccentricity of 20CAG eCNs decreases more sharply than that of the 72 CAG eCNs, but was no different than the *HTT* KO eCNs. **E)** The soma skew feature measures the symmetry gradient of pixels across the soma. The 20CAG eCNs changed at the same rate as the 72 CAG eCNs but changed at a different constant than the *HTT* KO eCNs. **F, G, H)** The gray level co-occurrence matrix (GLCM) features (GLCM Energy, Diss, Corr) all capture the texture of each object. All eCNs became smoother and less textured over time but at different constants. **I)** The histogram of gradients (HOG) feature captures complexity of structures and is similar to the edge feature, but captures pixels for the entire cell not just the edge. Over time, all groups of eCNs became less complex according to this feature, but at different rates. **J)** The Sholl median features captures the median number of processes emanating from each soma. The number of processes decreased over time in the control and 72CAG eCNs, whereas in the *HTT* KO eCNs the number of processes increased. **K)** The Minkowski-Bouligand feature is another complexity feature and also decreased over time in all groups, but at different rates. **L, M, N)** The Sobel operator features are additional edge detection features. These features decreased across all eCNs over time, but at different amounts across the groups. **O)** The Frangi feature, **P)** the Fast Fourier Transform (FFT) and **Q)** mean filter feature are measures of pixel pattern complexity, and similarly decreased over time for all groups, at different rates. All statistical analyses were conducted using RMedPower, and p-values were reported with corrections for multiple comparisons.

**Supplemental Figure 7: eCNs for OMIC analysis display similar cell type characterization.** At differentiation day 35 representative coverslips of omics eCN cultures were fixed and stained with antibodies directed at forebrain and early cortical neuron markers and imaged using fluorescence microscope. Images were subjected to a modified Cell Profiler pipeline as detailed in a previous study(Wang et al., 2024) to quantify the proportion of cell expressing each marker. A Linear Mixed Effects Model (LMM)(Kuznetsova et al., 2017; R Core Team, 2012) to quantify the percentage of cells positive for each antibody across each genotype group (20CAG control, expanded CAG (56CAG and 72CAG), and *HTT* KO) no outliers were removed. Significant differences between genotypes was only noted in FOXG1 expression with an increase in KO and expanded CAG lines compared to the controls, this is consistent with the transcriptomics data from these cultures, but not the proteomics data. **A).** Representative immunofluorescence images for all cell lines (20CAGn1, 20CAGn2, 56CAG, 72CAGn1, and *HTT* KO) show expression of neuronal forebrain markers FOXG1 (magenta), BCL-11B (green), TBR1 (magenta), mature neuronal marker MAP2 (magenta) and some remaining expression of immature neuronal marker NES (green) All cells stained with Hoescht (Blue) nuclear marker. Scale bar= 50um **B).**  Bar graphs showing the percent FOXG1 positive nuclei normalized to total nuclei per cell line (left) and per genotype (right) (n=12-16 images per cell line across 3 (4 for *HTT* KO) differentiations). The *HTT* KO shows increased expression compared to the control lines (β=0.795, p<5.97E-05) as do the expanded CAG lines (β=1.28, p<5.99E-06) with no significant difference between the expanded and KO lines (β=-0.463, p<0.378). **C).** Bar graphs showing the percent TBR1 positive nuclei normalized to total nuclei per cell line (left) and per genotype (right) (n=12-16 images per cell line across 3 (4 for HTTKO) differentiations). No significant differences are seen between the genotypes 20CAG vs *HTT* KO β=0.205, p<0.531, 20CAG vs expanded CAG β=0.411, p<0.378, expanded vs KO β=-0.235, p<0.614). **D).** Bar graphs showing the percent BCL11B positive nuclei normalized to total nuclei per cell line (left) and per genotype (right) (n=12-16 images per cell line across 3 (4 for *HTT* KO) differentiations). No significant differences are seen between the genotypes (20CAG vs *HTT* KO β=-0.519, p<0.531, 20CAG vs expanded CAG β=-0.489, p<0.531, expanded vs KO β=-0.039, p<0.927). Error bars represent standard deviation. Each dot represents the percent positive fraction of antibody- labeled cells measured from an entire image.

**Supplemental Figure 8: Pluripotent stage omics analysis. A)** PCA of pluripotent cell transcriptomics shows clustering by cell line across multiple replicates but not by HD status. Parentheses indicate percentage of variance explained by principal components 1 and 2. **B)** Number of differential genes comparing *HTT* KO to control, 56CAG to control and 72CAG to control in ES cells measured by ATAC-seq, H3K1 ChIP-seq, proteomics and transcriptomics. 56CAG is indicated in the gold bars, 72CAG is indicated in the gold bars and *HTT* KO is indicated in the purple bar. For the ATAC-seq and H3K4me1 ChIP-seq, we defined differential genes as genes for which there was at least one significant peak at FDR<0.1. **C)** Bar graph showing the normalized RNA expression levels of HTT in all cell lines from the pluripotent stage from RNAseq data. Dots represent replicates used in sequencing studies. **D)** A subnetwork from the integrative analysis of *HTT* KO multi-omic data in ES cells shows interactions between the down-regulated HTT protein with known its known interactor CREBBP, which in turn interacts with its known downstream effector FOS. Other interacting nodes such as the down-regulated protein TOP2A and FOXM1 are involved in DNA repair maintenance.

**Supplemental Figure 9: Primary Omics assays in ESC-derived cortical neurons**. **A)** Bar graph showing the normalized RNA expression levels of HTT in all cell lines from the cortical stage from RNAseq data. Dots represent replicates used in sequencing studies. **B)** PCA of H3K4me1 ChIP-seq shows partial separation by HD status along principal component 2. One replicate (20CAGn1, replicate 2) in the control in the H3K4me1 data was removed and excluded from downstream analysis. **C)** PCA of proteomics separates some cell lines by HD status along principal component 1. **D)** PCA of ATAC-seq separates some cell lines by HD status along principal component 2. One replicate (56CAG, replicate 3) in the ATAC-seq data was removed from the PCA for failing quality control benchmarks due to low read counts and was excluded from downstream analysis. **E)** PCA of H3K4me3 ChIP-seq separates some cell lines by HD status along principal component 1. **F)** PCA of H3K27me3 ChIP-seq separates some cell lines by HD status along principal component 2. **G)** PCA of H3K27Ac ChIP-seq separates some cell lines by replicate ID rather than genotype. Each genotype of the same replicate overlaps on the plot such that only the last genotype (red, *HTT* KO) is displayed. Parentheses in panels B-D and F-G indicate percentage of variance explained by principal components 1 and 2.

**Supplemental Figure 10: Comparison of transcriptional changes with previously published studies. A)** The differentially expressed genes between HD case and control transcriptomics significantly overlap with the differentially expressed genes between HD and control in from a previous study using iPSC-derived cortical neurons (hypergeometric p-value=2.0*10^-6^). **B)** The differentially expressed genes between HD case and control transcriptomics significantly overlap with previously published differentially expressed genes between HD and control from iPSC-derived striatal neurons (hypergeometric p-value<10^-16^). **C)** The differentially expressed genes between *HTT* KO and control have marginally significant overlap with the differentially expressed genes between HD and control in previously published iPSC-derived cortical neurons (p=0.02). **D)** The differentially expressed genes between *HTT* KO and control significantly overlap with differentially expressed genes between HD and control from a previously published study of iPSC-derived striatal neurons (p=3.8*10^-4^).

**Supplemental Figure 11: Summary of concordant changes analysis. A)** Differentially expressed genes between HD and control or *HTT* and control are enriched for genes with the same fold change across genotypes in each assay. UpSet plots of upregulated and downregulated genes show that genes that are concordant in fold change across multiple assays are among the largest intersection sets for **B)** upregulated genes and **C)** downregulated genes.

**Supplemental Figure 12: Degree of overlap between CAG expanded and KO networks: A)** The number of intersecting nodes between the *HTT* KO and HD networks are greater than expected by chance after measuring the Jaccard index of 1000 pairs of randomized networks. The Jaccard index between the *HTT* KO and HD networks is indicated by the dashed, vertical red line. **B)** The size of the largest connected component at the intersection of the *HTT* KO and HD network is larger than the largest connected component of any of 1000 other randomized networks. The percentage of nodes in the largest connected component is indicated by the dashed, vertical red line. **C)** Heatmap of the fold change between case and control in proteomics and transcriptomics for nodes that appear in the *HTT* KO network or the HD network. Many of these nodes have similar fold change directions across assays.

**Supplemental Figure 13: Unedited western for pluripotent-stage samples A)** Unedited blot stained with Revert total protein stain for ES stage samples (left) and the same blot labeled with a box indicating the area shown in Figure S1A (right). Samples not included (on the right side of the blot) were from an unrelated experiment that was also probed for HTT expression. Ladders shown are the LI-COR Chameleon Duo Ladder (left) and Tri-color Pre-stained Protein Marker II (Bioland Scientific). **B)** Unedited for ES stage samples probed with an N-terminal targeting HTT antibody (Abcam ab109115) (left) and the same blot labeled with a box indicating the area shown in Figure S1A (right). Samples not included (on the right side of the blot) were from an unrelated experiment that was also probed for HTT expression. Ladders shown are the LI-COR Chameleon Duo Ladder (left) and Tri-color Pre-stained Protein Marker II (Bioland Scientific).

**Supplemental Figure 14: Unedited western for cortical-stage samples A)** Unedited blot stained with Revert total protein stain for eCN samples (left) and the same blot labeled with a box indicating the area shown in Figure S1B (right). Samples not included (on the left side of the blot) are the ES stage samples re-run in figure S1A with additional protein loaded for better resolution. Ladder shown is the LI-COR Chameleon Duo Ladder (left). **B)** Unedited blot for eCN samples probed with an N-terminal targeting HTT antibody (Abcam ab109115) (left) and the same blot labeled with a box indicating the area shown in Figure S1B (right). Samples not included (on the left side of the blot) are the ES stage samples re-run in figure S1A with additional protein loaded for better resolution. Ladder shown is the LI-COR Chameleon Duo Ladder (left)

Cook, R. D. (1979). Influential Observations in Linear Regression. *Journal of the American Statistical Association*, *74*(365). <https://doi.org/10.2307/2286747>

Kuznetsova, A., Brockhoff, P. B., & Christensen, R. H. B. (2017). lmerTest package: Tests in linear mixed effects models. *J. Stat. Softw.*, *82*(13), 1–26. <https://doi.org/10.18637/jss.v082.i13>

R Core Team. (2012). *R: A language and environment for statistical computing*. R Foundation for Statistical Computing. Available from: <https://www.R-project.org/>.

Wang, B., Vartak, R., Zaltsman, Y., Naing, Z. Z. C., Hennick, K. M., Polacco, B. J., Bashir, A., Eckhardt, M., Bouhaddou, M., Xu, J., Sun, N., Lasser, M. C., Zhou, Y., McKetney, J., Guiley, K. Z., Chan, U., Kaye, J. A., Chadha, N., Cakir, M., . . . Krogan, N. J. (2024). A foundational atlas of autism protein interactions reveals molecular convergence. *BioRxiv*. <https://doi.org/10.1101/2023.12.03.569805>


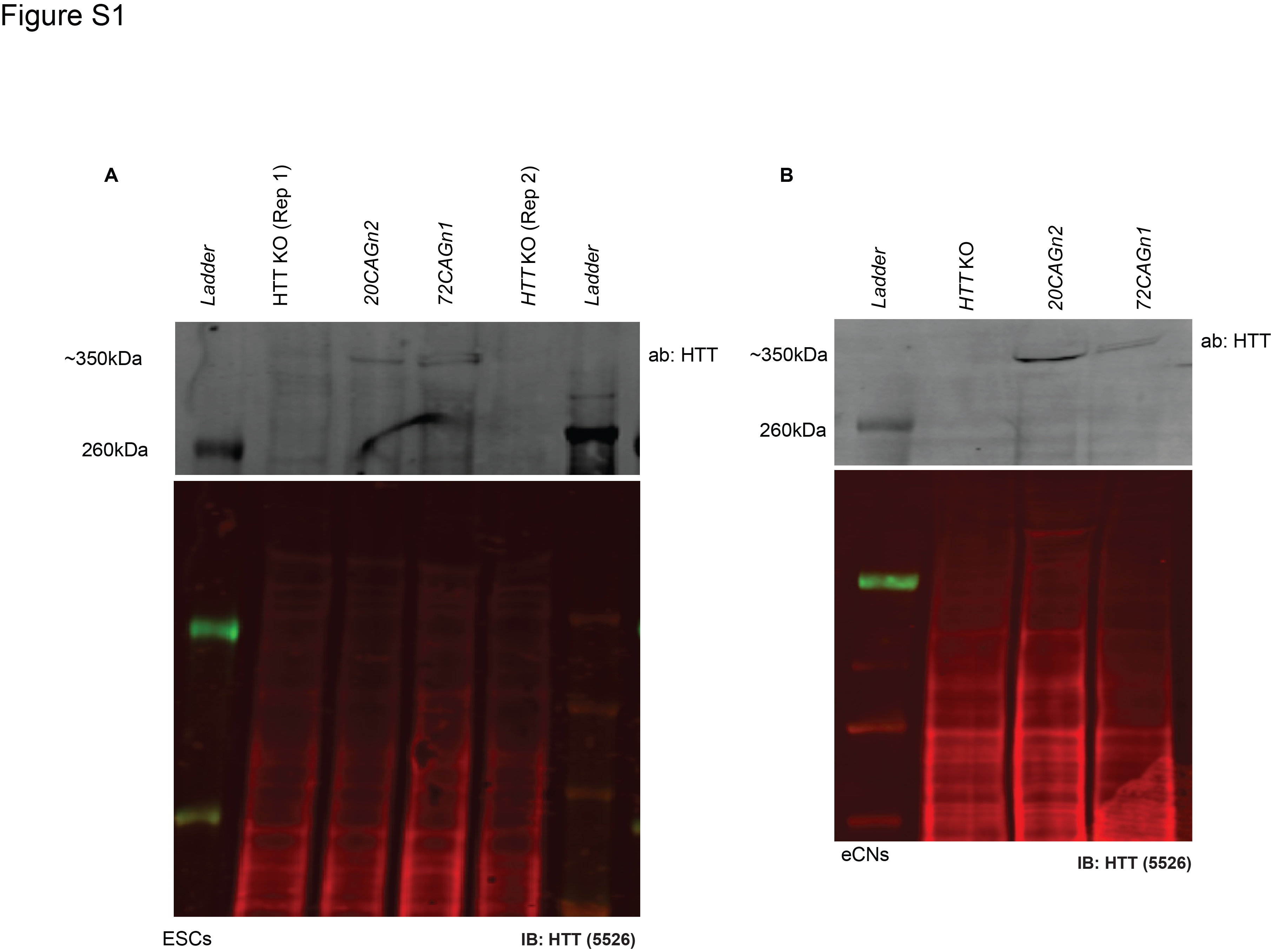


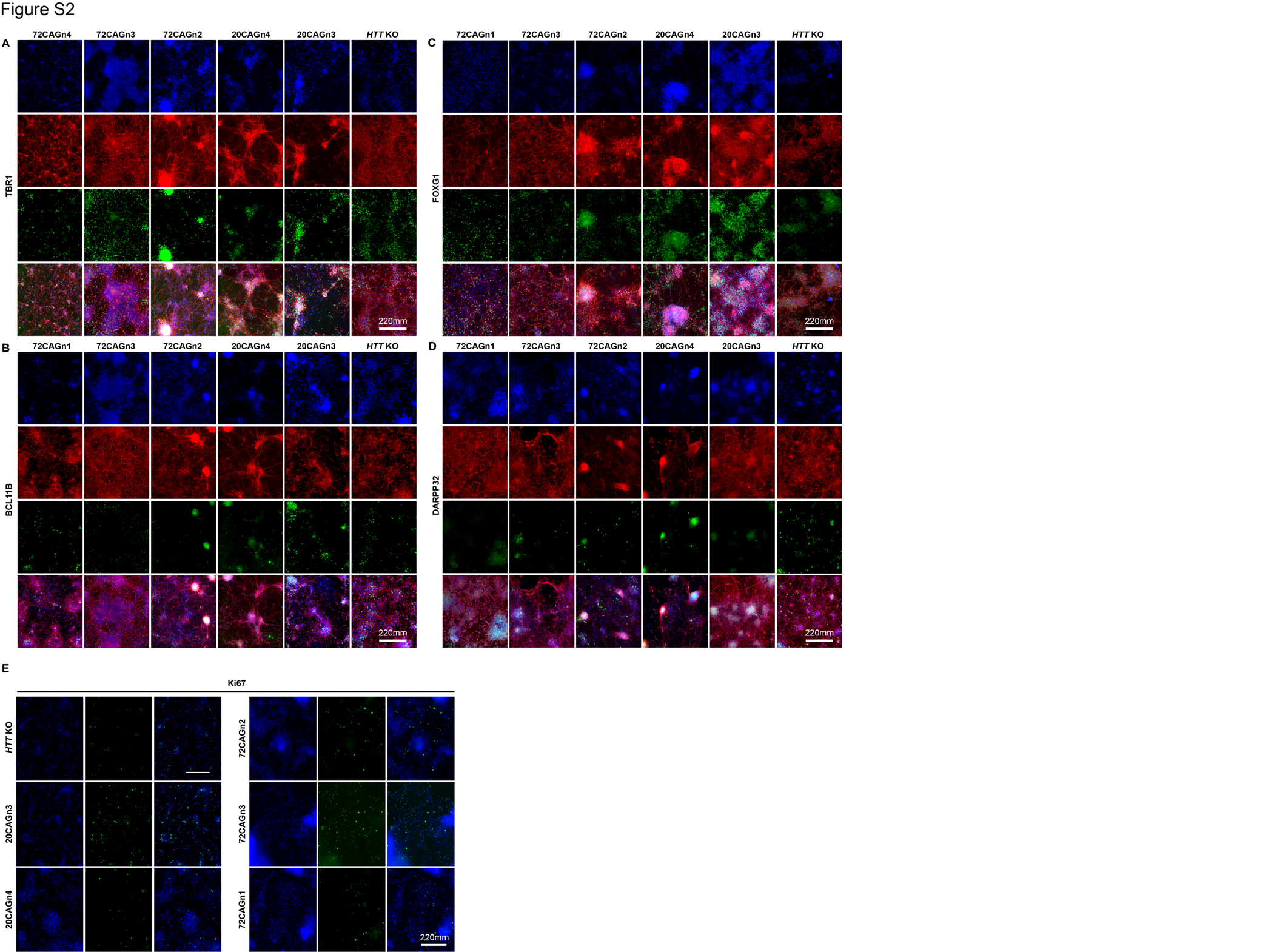


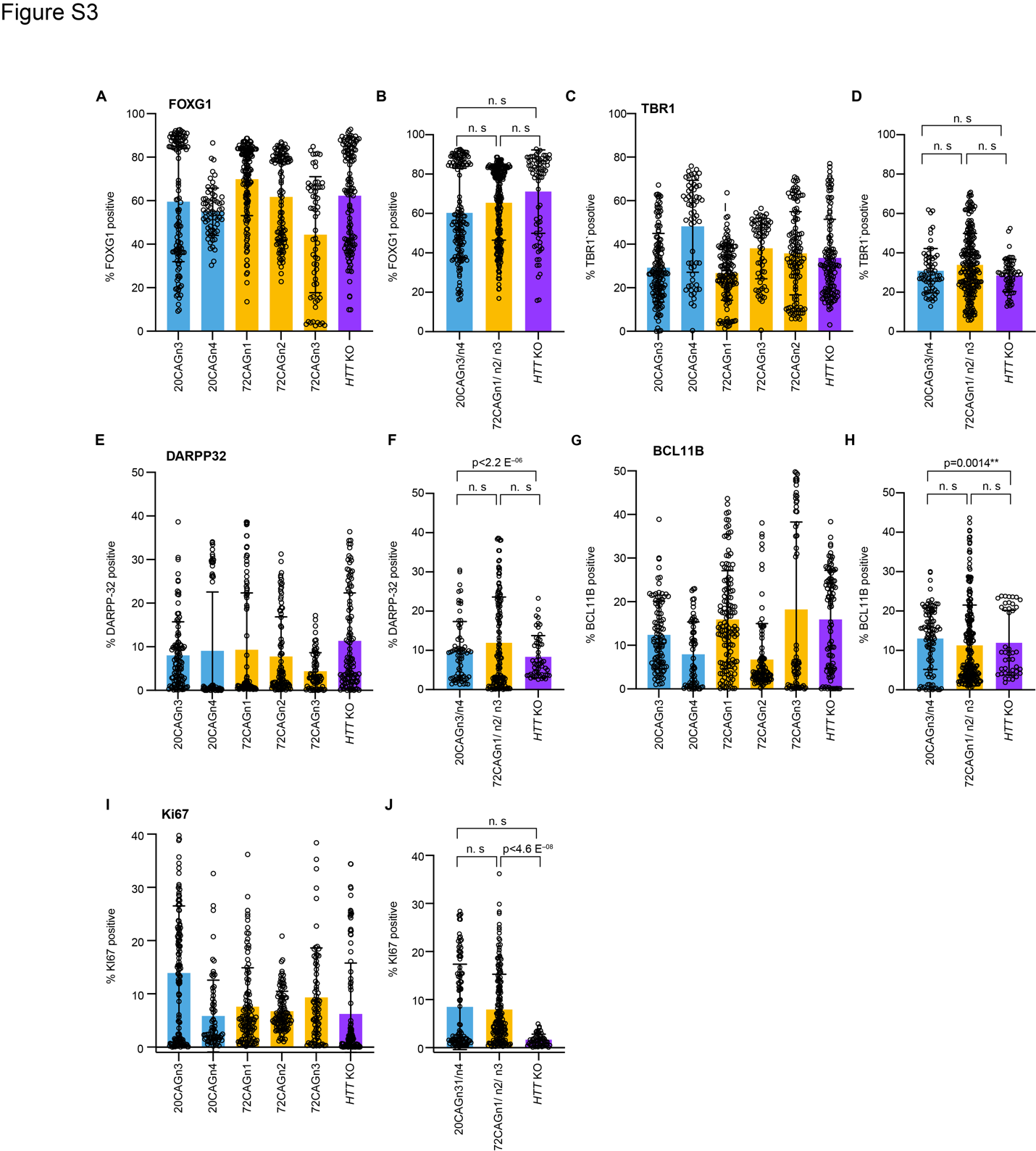


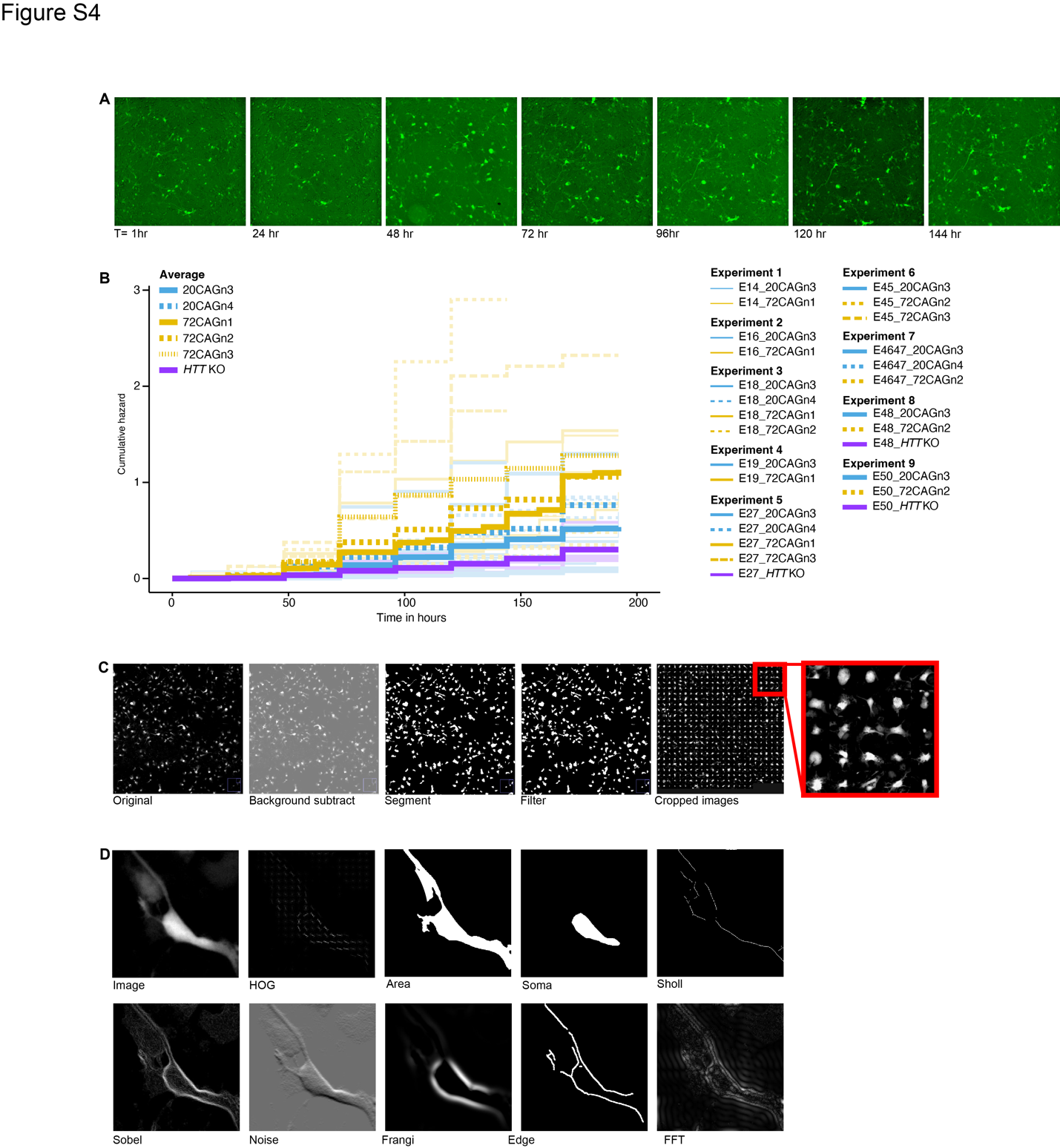


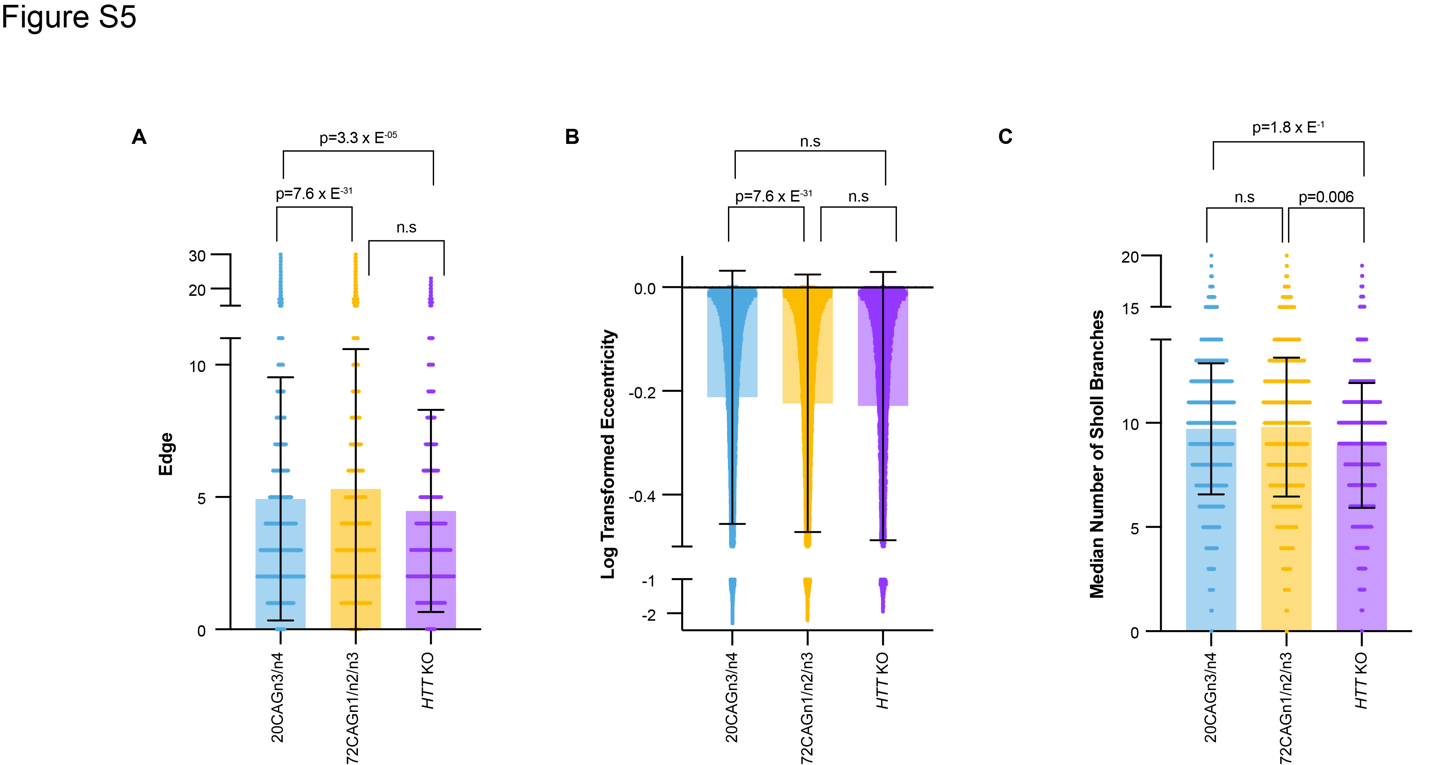


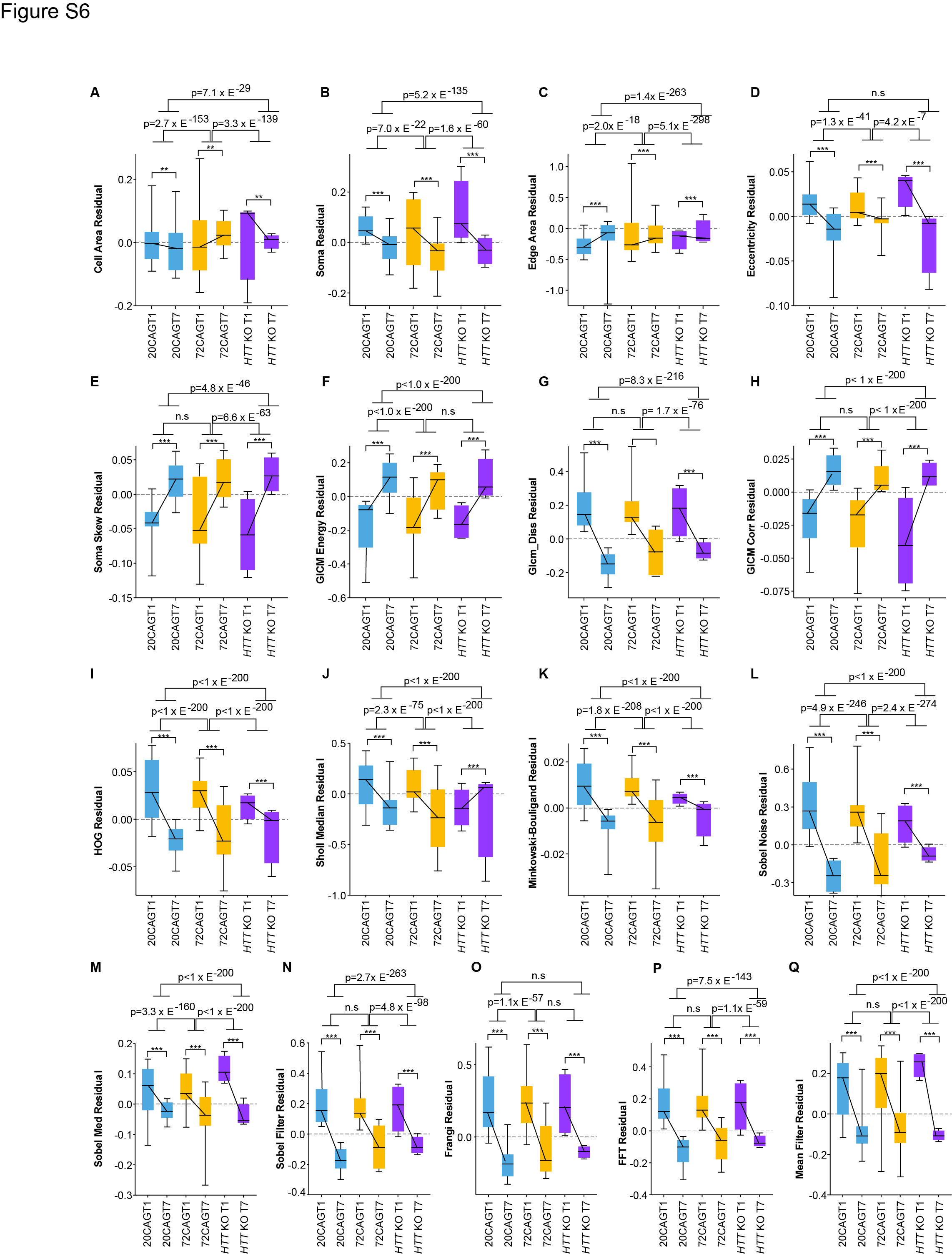


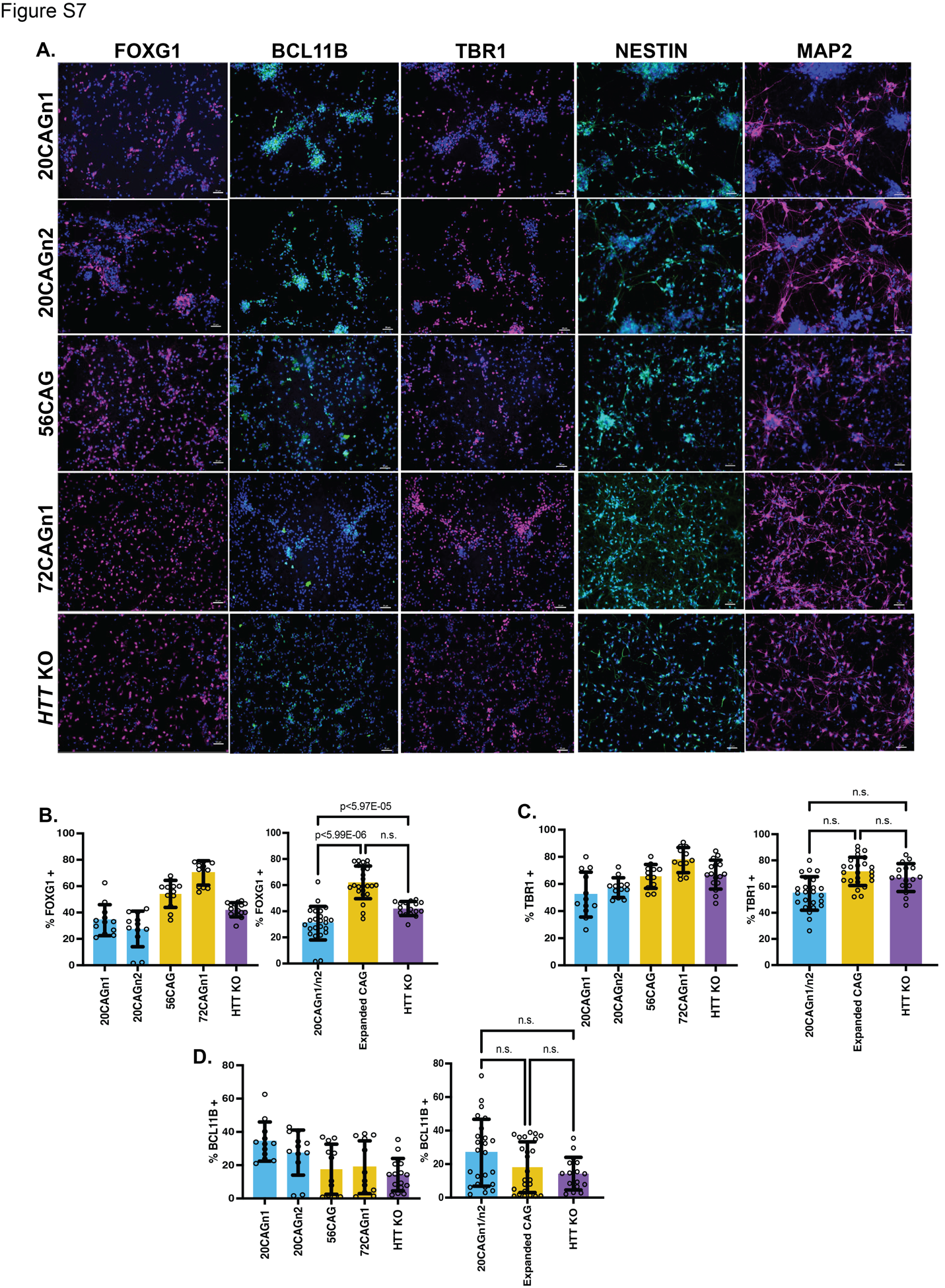


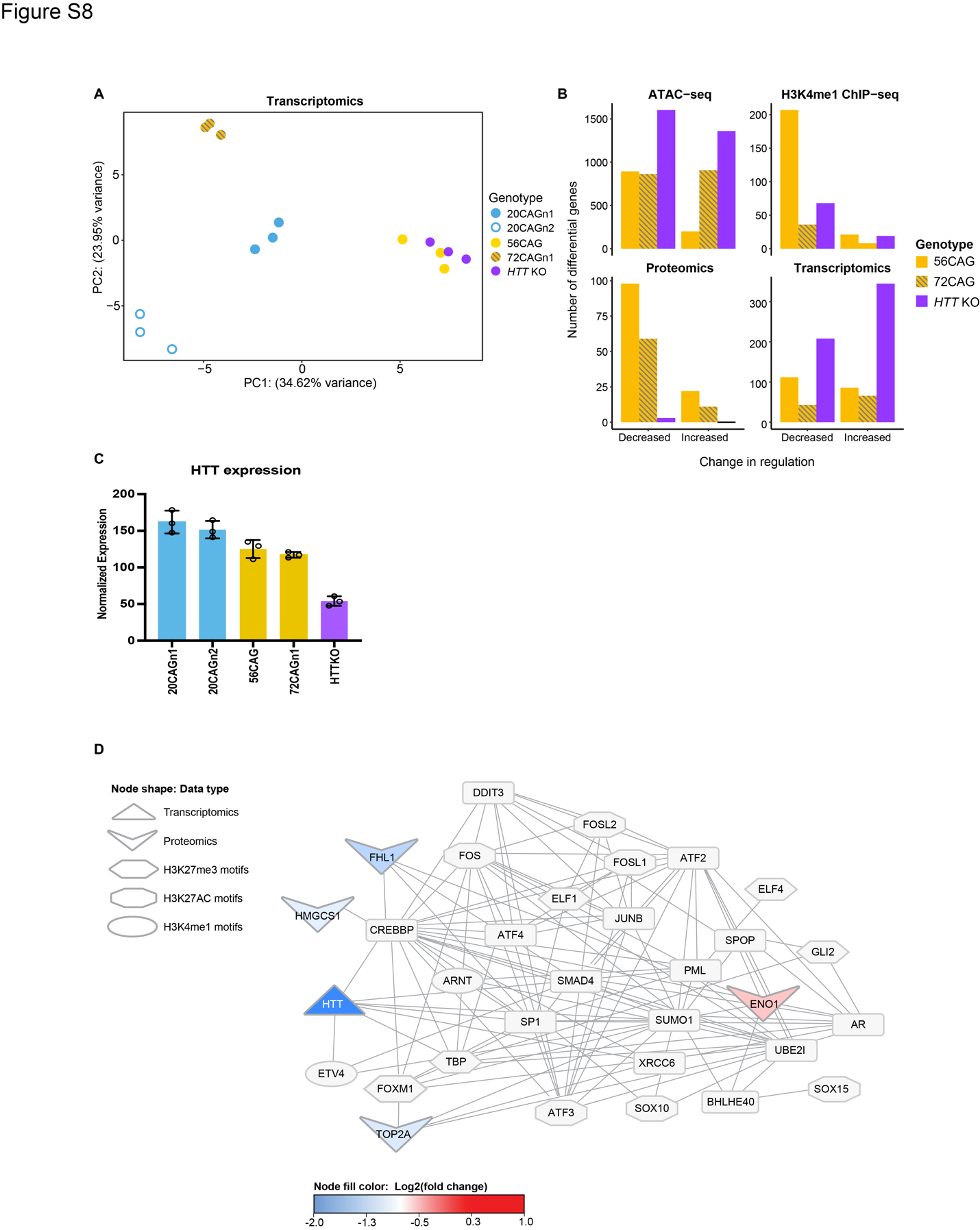


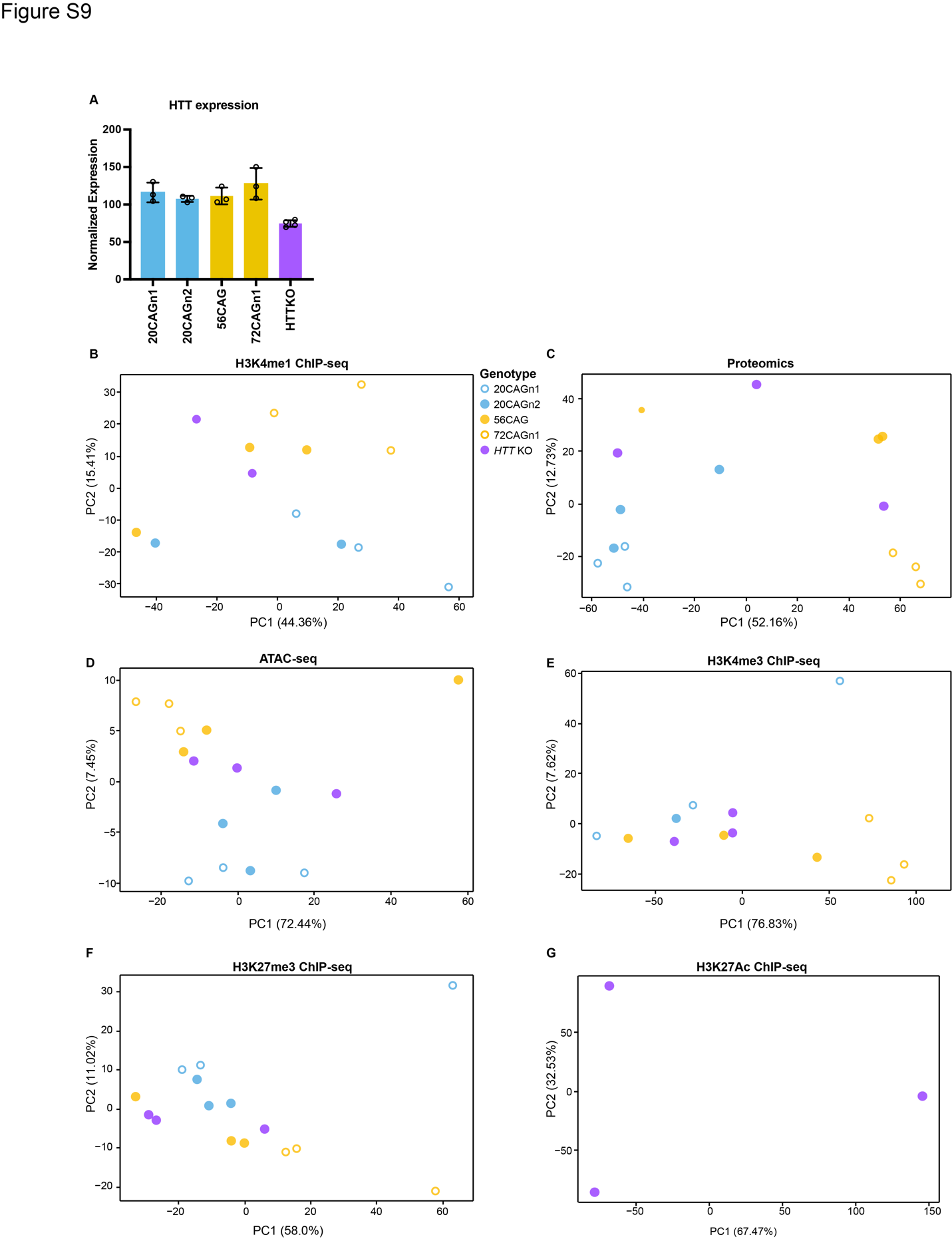


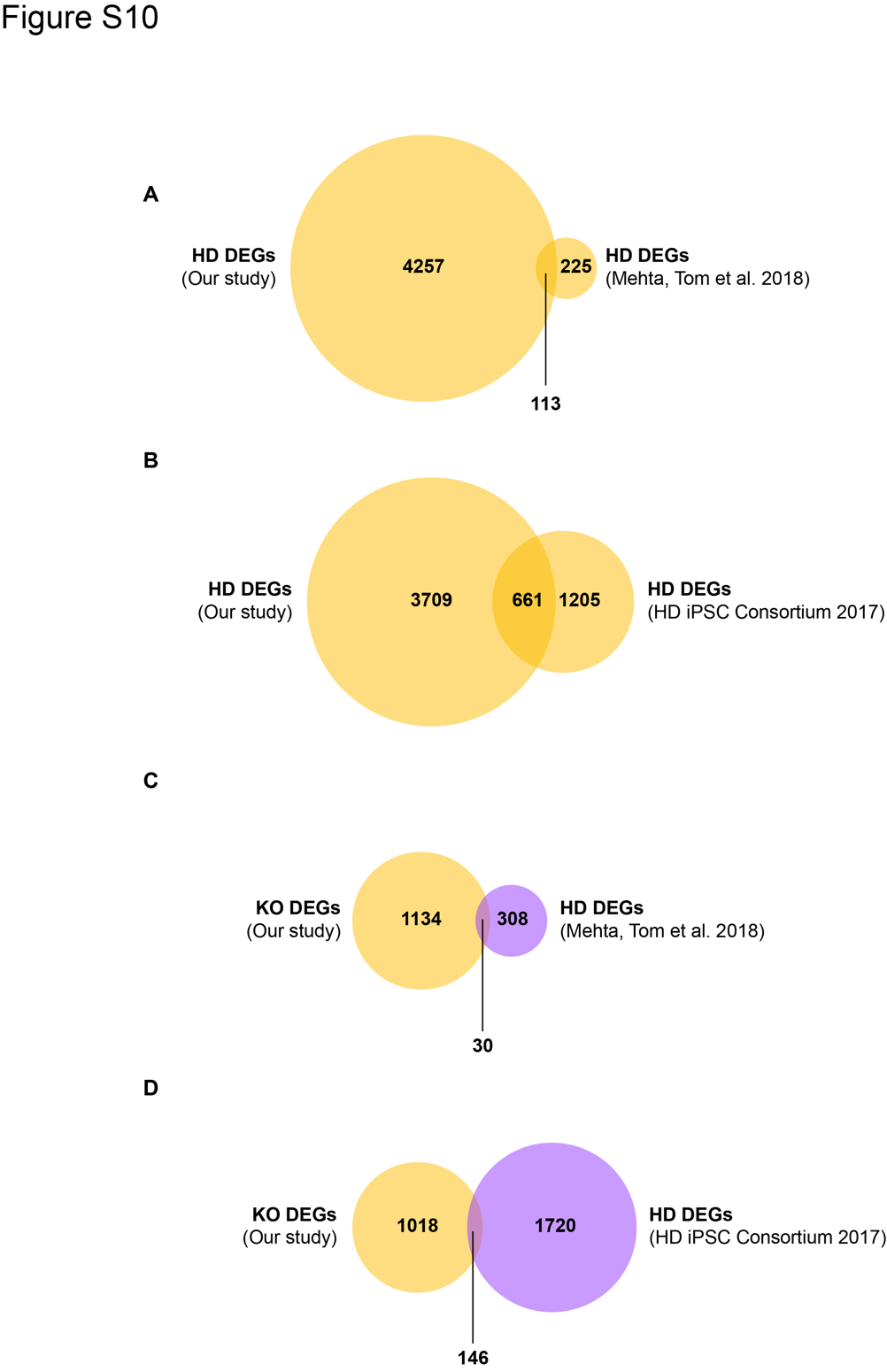


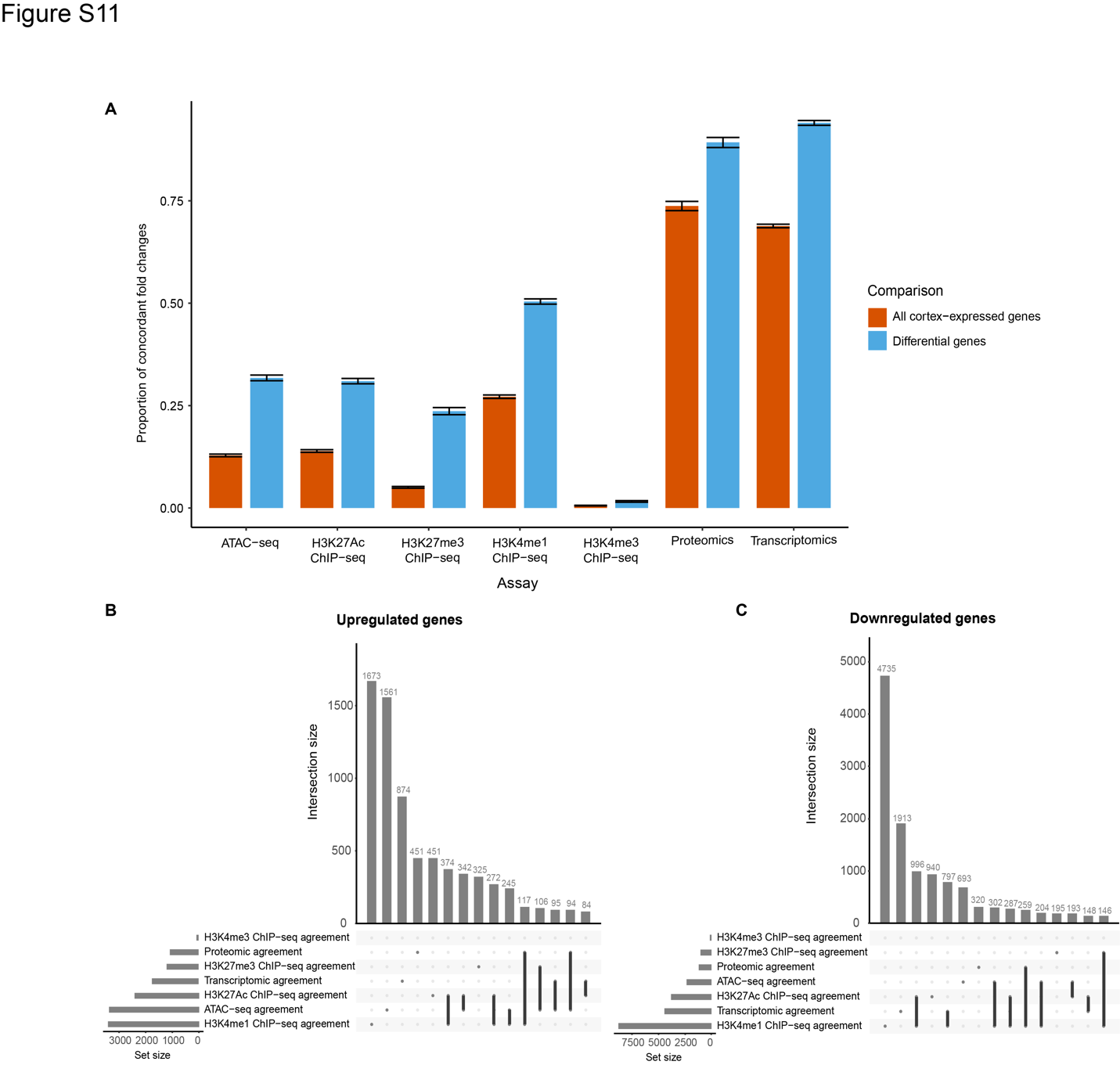


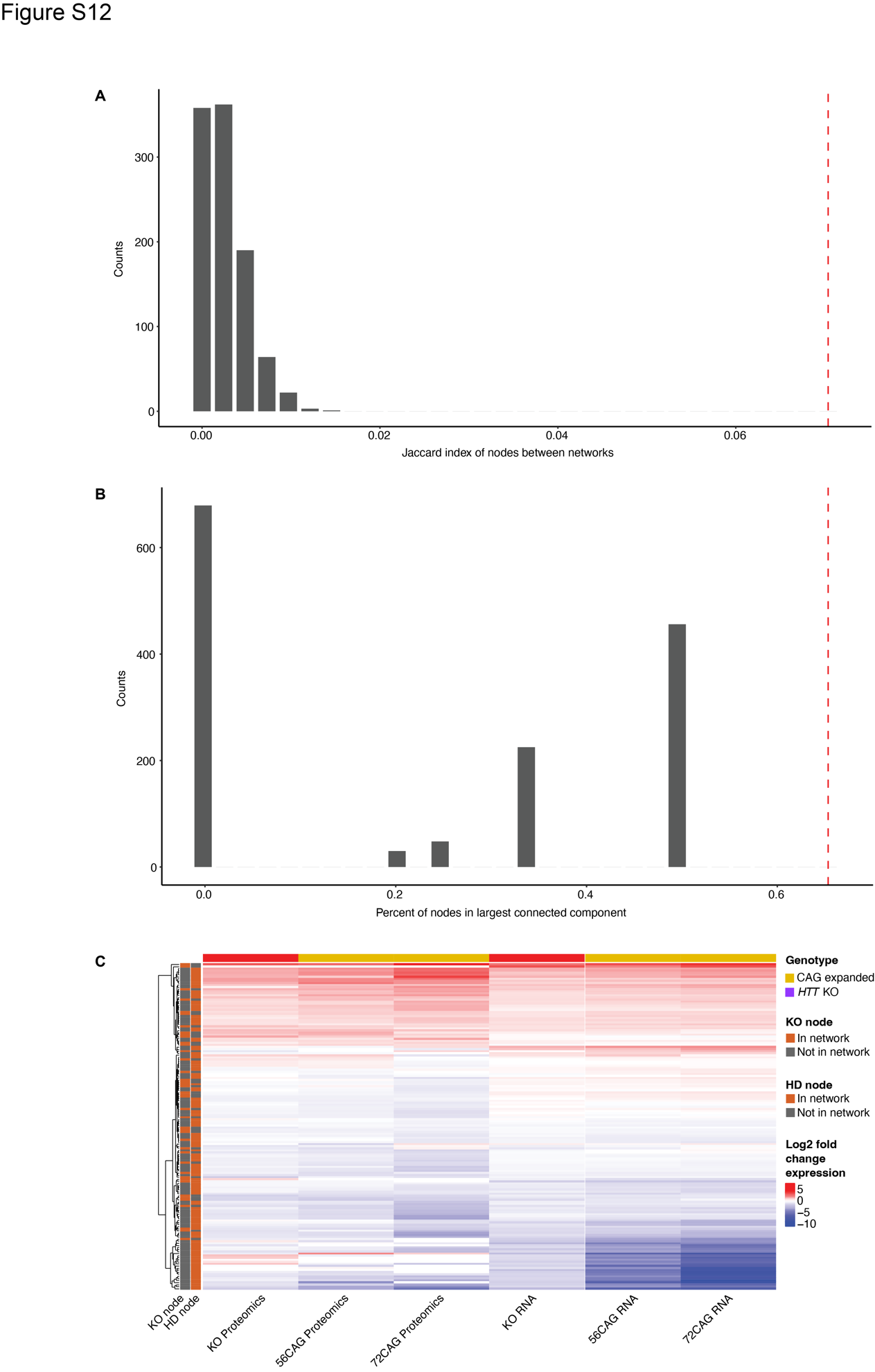


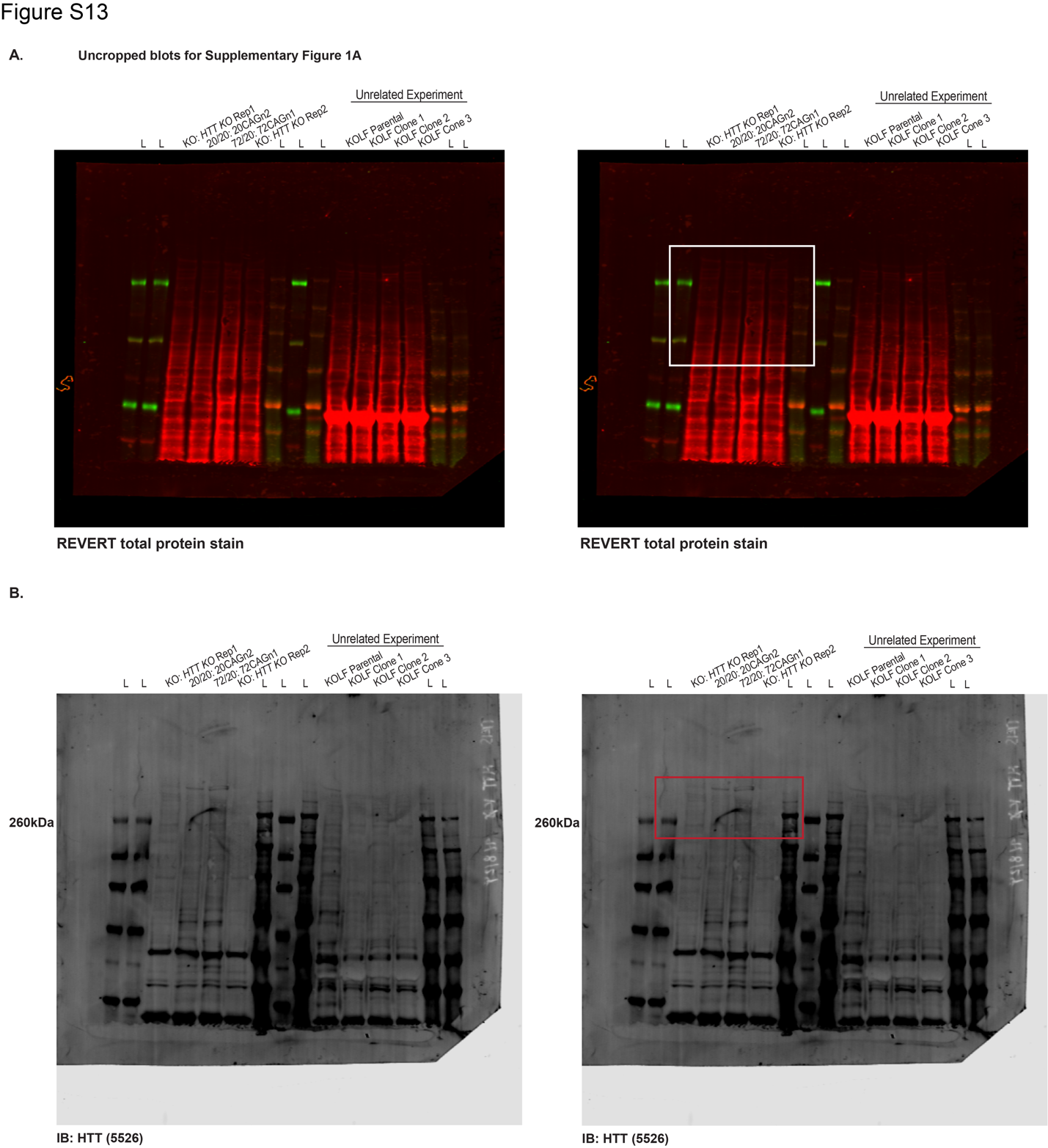


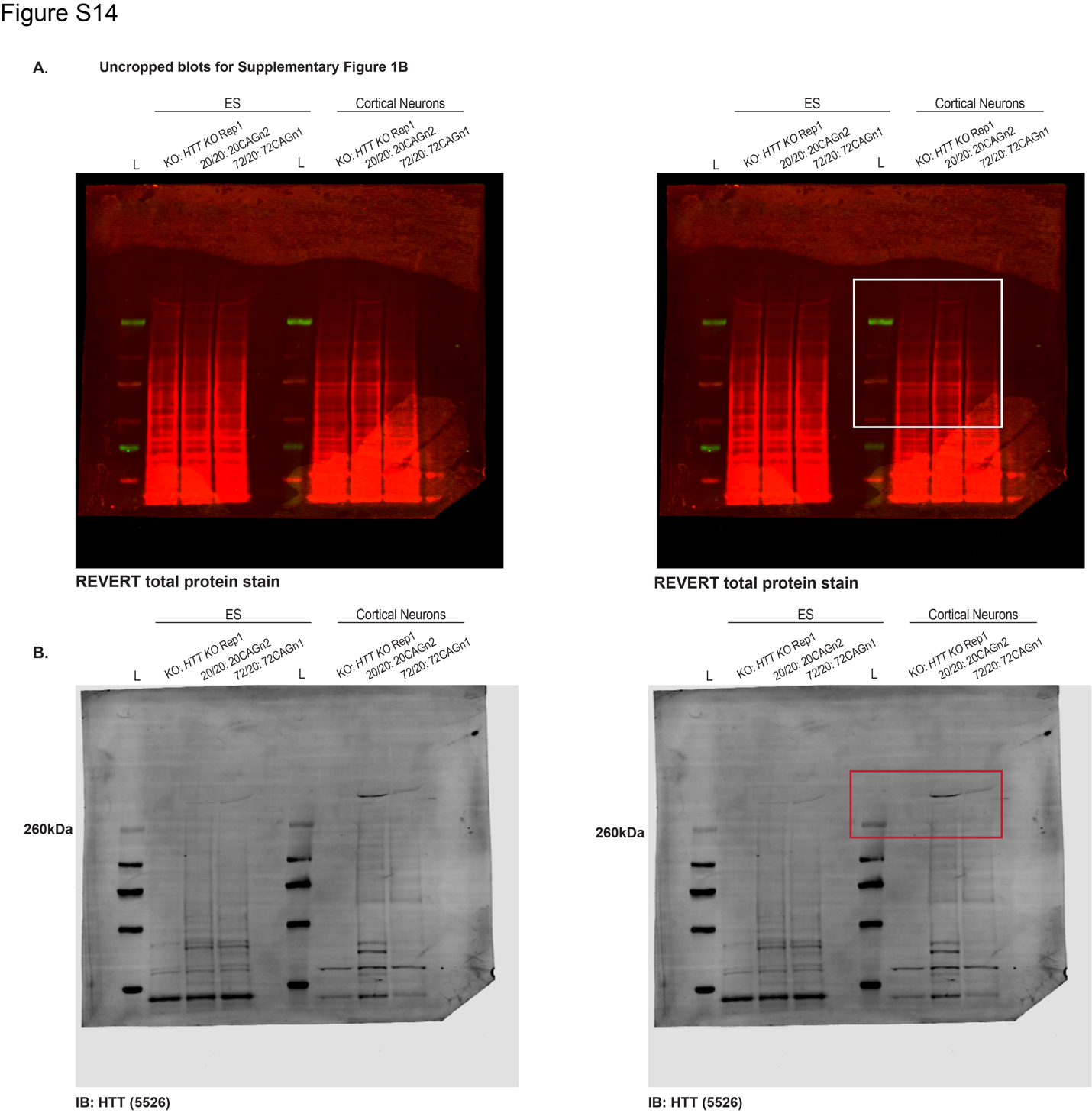

Supplement: 1 [file NIHMS2125198-supplement-1.docx]
