# Supplementary material for: Intersecting impact of CAG repeat and huntingtin knockout in stem cell-derived cortical neurons
Source: Neurobiol Dis. Author manuscript; Available in PMC 2025 Dec 1. (PMC12668206; doi:10.1016/j.nbd.2025.106914)
Supplement: 4 [file NIHMS2125198-supplement-4.docx]

Table S1: Table of cell lines and data collected in each cell line. Asterisk indicates unedited parental line. *(file mmc4)*

Table S2: Number of immunocytochemistry experiments performed, and the number of cells analyzed across experiments for each marker in each cell line. *(file mmc4)*

Table S3: Number of immunocytochemistry experiments performed, and the number of cells analyzed across experiments for each marker by genotype after outliers were removed. *(file mmc4)*

Table S4: Metadata relating to quality control metrics of pluripotent stage omics assays. *(separate excel file mmc2)*

Table S5: Metadata relating to quality control metrics of eCN stage omics assays. *(separate excel file mmc3)*

Table S6: Number of differential Omics at the ES stage relative to control ES lines. *(file mmc4)*

Table S7: Differential genes between *HTT* KO and control, 56CAG repeat expansion and control, and 72CAG repeat expansion and control in RNA-seq, proteomics, ChIP-seq and ATAC-seq data from ES cells. *(separate excel file mmc5)*

Table S8: List of the top 50 most variable genes for the eCN transcriptomics PCA analysis. *(file mm4)*

Table S9: List of the significant (p<0.05) Gene Ontology molecular function categories for the top 50 most variable genes from the eCN transcriptomics analysis. *(file mm4)*

Table S10: Number of differential Omics at the cortical neuron stage relative to control cell lines. *(file mm4)*

Table S11: Differential genes between *HTT* KO and control, 56CAG repeat expansion and control, and 72CAG repeat expansion and control in RNA-seq, proteomics, ChIP-seq, and ATAC-seq data from cortical neurons. *(separate excel file mmc6)*

Table S12: Nodes shared between both *HTT* KO and *HTT* CAG expanded repeat networks, as well as the fold changes of these genes relative to controls in RNA-seq, proteomics, ChIP-seq, and ATAC-seq data. *(separate excel file mmc7)*

Supplemental Table 1. Table of cell lines

| Line ID | CAG Repeat Alleles | CHDI ID | Line name | Assay |
| --- | --- | --- | --- | --- |
| 20CAGn1* | 22/20 | CHDI-90001539 | RUES2 | Omics |
| 20CAGn2 | 20/20 | CHDI-90002887 | RUES2_20(20)CAG-cl66 | Omics |
| 20CAGn3 | 22/20 | CHDI-90001585 | RUES2_20(22)CAG-cl30 | Imaging |
| 20CAGn4 | 22/20 | CHDI-90002034 | RUES2_20(22)CAG-cl65 | Imaging |
| 56CAG | 56/22 | CHDI-90001590 | RUES2_56(22)CAG-cl23 | Omics |
| 72CAGn1 | 72/20 | CHDI-90002877 | RUES2_72(20)CAG-cl12 | Omics/ Imaging |
| 72CAGn2 | 72/20 | CHDI-90003146 | RUES2_72(20)CAG-cl2 | Imaging |
| 72CAGn3 | 72/20 | CHDI-90003147 | RUES2_72(20)CAG-cl9 | Imaging |
| HTTKO | None | CHDI-90002878 | RUES2_Htt_KO-cl8A | OMICS/ Imaging |

*Unedited parental line

Supplemental Table 2.

| **Line** | **No. of Experiments** | | | | | **Total No. of Cells** | | | | |
| --- | --- | --- | --- | --- | --- | --- | --- | --- | --- | --- |
|  | **BCL11B** | **DARPP32** | **Ki67** | **FOXG1** | **TBR1** | **BCL11B** | **DARPP32** | **Ki67** | **FOXG1** | **TBR1** |
| **20CAGn3** | **6** | **5** | **6** | **5** | **6** | 157503 | 123259 | 165932 | 173957 | 208537 |
| **20CAGn4** | **3** | **3** | **3** | **3** | **3** | 61967 | 66514 | 88350 | 43933 | 6648 |
| **72CAGn1(12)** | **6** | **5** | **6** | **6** | **6** | 161264 | 116440 | 127997 | 183529 | 175079 |
| **72CAGn2(2)** | **5** | **4** | **5** | **5** | **5** | 215935 | 161215 | 187407 | 194541 | 223921 |
| **72CAGn3 (9)** | **3** | **3** | **4** | **3** | **3** | 101736 | 93563 | 113418 | 61064 | 95479 |
| **HTTKO** | **4** | **4** | **4** | **5** | **5** | 143054 | 127333 | 153447 | 142049 | 182798 |

Supplemental Table 3.

| **Line** | **No. of Experiments** | | | | | **Total No. of Cells** | | | | |
| --- | --- | --- | --- | --- | --- | --- | --- | --- | --- | --- |
|  | **BCL11B** | **DARPP32** | **Ki67** | **FOXG1** | **TBR1** | **BCL11B** | **DARPP32** | **Ki67** | **FOXG1** | **TBR1** |
| **20CAGn3/n4** | 5 | 4 | 5 | 5 | 4 | 136473 | 58726 | 136480 | 223881 | 98592 |
| **72CAGn1/2/3** | 7 | 6 | 7 | 7 | 4 | 369076 | 150327 | 255358 | 350443 | 194894 |
| **HTTKO** | 2 | 2 | 2 | 4 | 2 | 56836 | 59120 | 52029 | 70246 | 71805 |

Supplemental Table 6. ES stage quantification of differential genes and proteins

| Cell type | Group | Count | Assay | Direction |
| --- | --- | --- | --- | --- |
| ES | 56CAG | 86 | Transcriptomics | Increased |
| ES | 56CAG | 112 | Transcriptomics | Decreased |
| ES | 72CAG | 66 | Transcriptomics | Increased |
| ES | 72CAG | 43 | Transcriptomics | Decreased |
| ES | HTT KO | 345 | Transcriptomics | Increased |
| ES | HTT KO | 208 | Transcriptomics | Decreased |
| ES | HTT KO | 0 | Proteomics | Increased |
| ES | HTT KO | 3 | Proteomics | Decreased |
| ES | 56CAG | 22 | Proteomics | Increased |
| ES | 56CAG | 98 | Proteomics | Decreased |
| ES | 72CAG | 11 | Proteomics | Increased |
| ES | 72CAG | 59 | Proteomics | Decreased |
| ES | HTT KO | 1359 | ATAC-seq | Increased |
| ES | HTT KO | 1602 | ATAC-seq | Decreased |
| ES | 56CAG | 200 | ATAC-seq | Increased |
| ES | 56CAG | 892 | ATAC-seq | Decreased |
| ES | 72CAG | 907 | ATAC-seq | Increased |
| ES | 72CAG | 862 | ATAC-seq | Decreased |
| ES | HTT KO | 19 | H3K4me1 ChIP-seq | Increased |
| ES | HTT KO | 68 | H3K4me1 ChIP-seq | Decreased |
| ES | 56CAG | 21 | H3K4me1 ChIP-seq | Increased |
| ES | 56CAG | 207 | H3K4me1 ChIP-seq | Decreased |
| ES | 72CAG | 8 | H3K4me1 ChIP-seq | Increased |
| ES | 72CAG | 36 | H3K4me1 ChIP-seq | Decreased |

Supplemental Table 8. Top 50 most variable genes

| MPZL2 | EPYC | PRRX1 | LUM | FBLN2 | TBX15 | NFIX |
| --- | --- | --- | --- | --- | --- | --- |
| PCSK9 | RBM47 | FGFR2 | PDGFRB | TTR | IFITM1 | SFRP2 |
| SULT1E1 | COLEC12 | FGFR3 | CPED1 | CAVIN1 | CRYM |  |
| ALX4 | TWIST1 | PGM5 | HAPLN1 | AHNAK | PRRX2 |  |
| THBS1 | ID3 | TIMP3 | WNT8B | MYL9 | PMP22 |  |
| CDC20B | FOXC1 | SDC4 | SMAD6 | ERG | PDGFRA |  |
| GJA1 | PRSS35 | ID1 | ASPN | LGALS1 | RGCC |  |
| MSX2 | COL2A1 | TMEM119 | COL1A2 | COL3A1 | PRDM6 |  |

Supplemental Table 9. Gene Ontology Molecular Function of top 50 most variable genes

| **GO Molecular Function** | **Term_ID** | **p_adj** | **Genes** |
| --- | --- | --- | --- |
| platelet-derived growth factor binding | GO:0048407 | 1.44E-08 | COL2A1,PDGFRB,COL1A2,  COL3A1,PDGFRA |
| extracellular matrix structural constituent | GO:0005201 | 2.8731E-06 | THBS1,COL2A1,LUM,HAPLN1,  ASPN,COL1A2,FBLN2,COL3A1 |
| low-density lipoprotein particle binding | GO:0030169 | 0.00339519 | PCSK9,THBS1,COLEC12 |
| transmembrane receptor protein tyrosine kinase activity | GO:0004714 | 0.00756641 | FGFR2,FGFR3,PDGFRB,  PDGFRA |
| fibroblast growth factor binding | GO:0017134 | 0.01060978 | THBS1,FGFR2,FGFR3 |
| transcription regulator inhibitor activity | GO:0140416 | 0.01210479 | ID3,ID1,SMAD6 |
| SMAD binding | GO:0046332 | 0.017642 | SMAD6,COL1A2,COL3A1,  RGCC |
| fibronectin binding | GO:0001968 | 0.02166764 | THBS1,SDC4,SFRP2 |
| thyroid hormone binding | GO:0070324 | 0.03973589 | TTR,CRYM |

Supplemental Table 10. Quantification of differential genes and proteins

| Cell Type | Group | Assay | Differential Counts | Genotype |
| --- | --- | --- | --- | --- |
| Cortical Neurons | Decreased | H3K4me1 ChIP-seq | 8249 | 56/22 |
| Cortical Neurons | Increased | H3K4me1 ChIP-seq | 4760 | 56/22 |
| Cortical Neurons | Decreased | ATAC-seq | 4604 | 56/22 |
| Cortical Neurons | Increased | ATAC-seq | 7072 | 56/22 |
| Cortical Neurons | Decreased | Proteomics | 341 | 56/22 |
| Cortical Neurons | Increased | Proteomics | 233 | 56/22 |
| Cortical Neurons | Decreased | Transcriptomics | 3864 | 56/22 |
| Cortical Neurons | Increased | Transcriptomics | 1382 | 56/22 |
| Cortical Neurons | Decreased | H3K4me1 ChIP-seq | 10728 | 72/20 |
| Cortical Neurons | Increased | H3K4me1 ChIP-seq | 5172 | 72/20 |
| Cortical Neurons | Decreased | ATAC-seq | 5953 | 72/20 |
| Cortical Neurons | Increased | ATAC-seq | 10634 | 72/20 |
| Cortical Neurons | Decreased | Proteomics | 1235 | 72/20 |
| Cortical Neurons | Increased | Proteomics | 999 | 72/20 |
| Cortical Neurons | Decreased | Transcriptomics | 4022 | 72/20 |
| Cortical Neurons | Increased | Transcriptomics | 1476 | 72/20 |
| Cortical Neurons | Decreased | H3K4me1 ChIP-seq | 4278 | KO |
| Cortical Neurons | Increased | H3K4me1 ChIP-seq | 3321 | KO |
| Cortical Neurons | Decreased | ATAC-seq | 3175 | KO |
| Cortical Neurons | Increased | ATAC-seq | 5828 | KO |
| Cortical Neurons | Decreased | Proteomics | 133 | KO |
| Cortical Neurons | Increased | Proteomics | 84 | KO |
| Cortical Neurons | Decreased | Transcriptomics | 739 | KO |
| Cortical Neurons | Increased | Transcriptomics | 425 | KO |
